# Challenges and Future Perspectives in Modeling Neurodegenerative Diseases Using Organ‐on‐a‐Chip Technology

**DOI:** 10.1002/advs.202403892

**Published:** 2024-06-23

**Authors:** Francesca Michela Pramotton, Sarah Spitz, Roger D. Kamm

**Affiliations:** ^1^ Department of Mechanical Engineering and Biological Engineering Massachusetts Institute of Technology Cambridge MA 02139 USA

**Keywords:** brain‐on‐chip, microphysiological systems, neurodegenerative diseases, organ‐on‐a‐chip

## Abstract

Neurodegenerative diseases (NDDs) affect more than 50 million people worldwide, posing a significant global health challenge as well as a high socioeconomic burden. With aging constituting one of the main risk factors for some NDDs such as Alzheimer's disease (AD) and Parkinson's disease (PD), this societal toll is expected to rise considering the predicted increase in the aging population as well as the limited progress in the development of effective therapeutics. To address the high failure rates in clinical trials, legislative changes permitting the use of alternatives to traditional pre‐clinical in vivo models are implemented. In this regard, microphysiological systems (MPS) such as organ‐on‐a‐chip (OoC) platforms constitute a promising tool, due to their ability to mimic complex and human‐specific tissue niches in vitro. This review summarizes the current progress in modeling NDDs using OoC technology and discusses five critical aspects still insufficiently addressed in OoC models to date. Taking these aspects into consideration in the future MPS will advance the modeling of NDDs in vitro and increase their translational value in the clinical setting.

## Graphical abstract

Neurodegenerative diseases (NDDs) affect over 50 million globally, posing a growing health and economic burden. Traditional in vivo models have high failure rates in clinical trials. Organ‐on‐a‐chip (OoC) technology offers a promising alternative, closely mimicking human tissue niches in vitro. This review discusses OoC's progress in NDD modeling and identifies five critical areas needing further development to increase clinical translation.

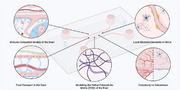

## 1 Introduction

Characterized by progressive degeneration of neurons, NDDs encompass a group of debilitating disorders impacting millions of people worldwide. The primary risk factor for many NDDs, including AD and PD, is aging.^[^ ^1^ ^]^ With a prevalence of 6.7 million people in the United States alone AD constitutes the most common NDD, affecting approximately one in ten individuals aged 65 years and older.^[^ ^2^ ^]^ Despite their distinct manifestations, prevalent NDDs, including AD, PD, amyotrophic lateral sclerosis (ALS), and Huntington's disease (HD), share a range of common phenotypes, including neuronal loss, gliosis, neuroinflammation, oxidative stress, mitochondrial dysfunction, and vascular damage. The precise mechanisms triggering NDD onset remain elusive, however, prevailing hypotheses implicate dysfunctional proteins, including amyloid β (aβ), α‐synuclein (α‐syn), and TDP‐43, as pivotal players.^[^ ^3^ ^]^ While significant strides have been made in the last few years in unraveling critical underlying mechanisms governing NDDs, this progress is just beginning to be effectively translated into clinical practice. This holds particular significance considering the substantial socio‐economic burden associated with NDDs, which is expected to surge significantly alongside the aging population, predicted to double by 2050.^[^ ^4^ ^]^ Projections indicate that cases of dementia, an umbrella term used to describe conditions associated with impaired cognitive functions and behavioral deficits, are estimated to triple, reaching 153 million by 2050.^[^ ^5^ ^]^ Despite these discouraging projections, a handful of drugs, capable of reducing the rate of disease progression, have received regulatory approval in recent years, including Aducanumab (2021) and Lecanemab (2023) for AD and Riluzole (2022) and Edaravone (2017) for ALS.^[^ ^6^ ^]^

Low success rates in clinical trials can be attributed at least in part, to the inability of current pre‐clinical animal models to adequately replicate NDD‐associated phenotypes.^[^ ^7^ ^]^ To that end, there is an increasing demand for pre‐clinical models that more closely mimic or predict human (patho)physiology. Recognizing this need, the US Food and Drug Administration (FDA) recently authorized the use of alternative modeling strategies in clinical studies by removing the requirement for animal testing as part of the FDA Modernization Act 2.0.^[^ ^8^ ^]^ One approach that has been deemed particularly suitable for pre‐clinical use is OoC technology.^[^ ^9^ ^]^ While OoC technology has not yet been widely used for drug development or screening, pharmaceutical companies have begun incorporating OoC data in their FDA submissions for regulatory approval.^[^ ^10^, ^11^ ^]^ OoC platforms refer to in vitro culture systems designed to emulate tissue or organ‐specific features by exposing cells to (patho)physiological microenvironments or stimuli. Building on microfluidic principles, OoC technology offers unique advantages over conventional in vitro culture systems including precise control over cellular arrangement, spatiotemporal cues as well as biomechanical stimulation.^[^ ^12^ ^]^ Employing OoC technology, significant advances have been made in replicating microphysiological tissue niches, including that of the brain in vitro, in recent years.^[^ ^13^, ^14^, ^15^, ^16^, ^17^, ^18^, ^19^ ^]^

**Figure 1:**
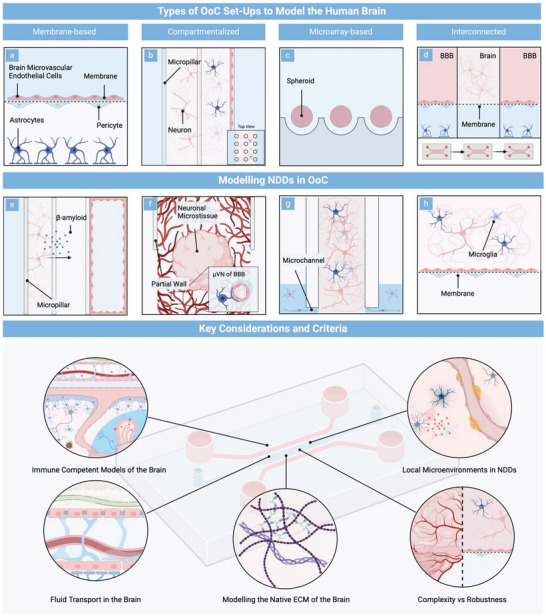
Top panel: Schematic representation of the cross‐sectional areas of the four different types of microfluidic setups employed in modeling the human brain, including a) membrane‐based, b) compartmentalized, c) microarray‐based, and d) interconnected platforms. Schematic representation of the cross‐sectional areas of critical MPS setups that have been employed to model NDDs in vitro, including e) a compartmentalized platform to model the effect of aβ on an endothelial lumen, f) a neurovascular co‐culture model interconnecting aβ overexpressing neurospheres with a self‐assembled network of the BBB, g) a compartmentalized AD platform for emulating microglial recruitment, and (h) a membrane‐based model to study vascular dysfunction in the context of PD. Bottom panel: Graphical representation of the key considerations and criteria MPS developers need to address prior to advancing microphysiological NDD models.

In general, one can distinguish between four approaches to modeling the human brain or its constituents in vitro, including membrane‐based i), compartmentalized ii), microarray‐based iii), and interconnected iv) setups. Membrane‐based microfluidic setups (**Figure** **1**a) constitute the most common methodology for modeling physiological barriers in OoC technology. This approach, inspired by the traditional Transwell protocol, employs a porous membrane to physically separate microvascular endothelial cells from astrocytes and pericytes, thereby replicating the luminal and abluminal compartments of the blood–brain barrier (BBB) in vitro. Physiological mimicry can be increased by incorporating additional cell (sub‐)types of the neurovascular unit. For example, to enhance cellular heterogeneity within their microfluidic model, *Vatine* et al.^[^ ^20^ ^]^ introduced dissociated pre‐rosette neural progenitor cells into the abluminal compartment of their platform, giving rise to a mixed population of induced pluripotent stem cell (iPSC)‐derived astrocytes, neurons as well as neural progenitor cells. Compartmentalized platforms, the most common microfluidic approach (Figure 1b), utilize microfabricated structures such as partial walls, micropillars, or microchannels to introduce distinct chambers within a microfluidic device. To illustrate, *Adriani* et al.^[^ ^21^ ^]^ employed an OoC platform comprised of four channels, separated by three micropillar arrays arranged in parallel to culture neurons and astrocytes side‐by‐side embedded within a collagen I hydrogel adjacent to an endothelial lumen. Similarly, microarrays (Figure 1c) utilize micrometer‐sized elements, e.g., microwells or micropillars, to physically restrict defined numbers of cells, guiding controlled cellular aggregation. This technique, characterized by high parallelization and ease of use, constitutes the most prevalent culture strategy among neuronal microtissues in OoC technology. For instance, *Zhu* et al.^[^ ^22^ ^]^ reported a micropillar array for the medium‐scale generation of iPSC‐derived cerebral organoids. Improving inter‐pillar distances and adjusting pillar diameters led to the generation of homogeneous organoid populations with uniform morphologies and sizes. The fourth approach to emulating constituents of the brain in vitro aims at enhancing model complexity via interconnecting modular microfluidic elements (Figure 1d). For example, *Maoz* et al.^[^ ^23^ ^]^ connected three membrane‐based devices in series to replicate influx into and efflux out of the brain parenchyma across the BBB. In addition to improved biomimicry, the authors demonstrated metabolic coupling between iPSC‐derived neuronal populations and the BBB, underscoring the importance of increased cellular complexity in (patho)physiological modeling.

OoC technology has introduced a set of microfluidic modeling strategies capable of emulating intricate tissue niches of the human brain in vitro, leading to considerable advances in modeling NDDs in recent years. *Park* et al.,^[^ ^24^ ^]^ for example, investigated the effects of low interstitial fluid flow on aβ_42_‐mediated neurotoxicity using a multi‐sphere array connected to an osmotic pressure‐driven pump. Dynamic exposure to aβ_42_ resulted in axonal degeneration, synaptic dysfunction, and neuronal death; neurotoxic effects were exacerbated under interstitial fluid flow. *Shin* et al.^[^ ^19^ ^]^ employed a compartmentalized microfluidic setup to co‐culture genetically modified neural progenitor cells with familial AD mutations adjacent to a single endothelial lumen (Figure 1e). The model emulated key pathological phenotypes, including impaired barrier integrity characterized by a decreased expression of claudin‐1, claudin‐5, and VE‐cadherin, as well as increased expression of matrix‐metalloproteinase‐2 and reactive oxygen species (ROS). Furthermore, the authors reported aβ deposition close to the endothelial lumen after six days of co‐culture, a common phenotype associated with cerebral amyloid angiopathy. Building on these original findings, *Ko* et al.,^[^ ^25^ ^]^ formed a perfusable microvascular network of the BBB adjacent to pre‐differentiated neuronal microtissues (Figure 1f) derived from the same progenitor source. Using this setup, the authors replicated key pathological phenotypes in vitro, including morphological aberrations within the vascular network, impaired barrier permeability, as well as abluminal aβ deposition after seven days of microfluidic co‐culture. To model microglial recruitment in the context of AD, *Park* et al.^[^ ^26^ ^]^ developed a concentrically arranged microfluidic platform comprised of two chambers interconnected by radially organized microchannels (Figure 1g). Using this tri‐culture system, the authors replicated critical phenotypes of the NDD in vitro, encompassing microglial recruitment, aβ aggregation, phosphorylated tau (p‐tau) accumulation as well as neuroinflammatory activity.

Progress in emulating NDD‐associated pathological phenotypes utilizing OoC also extends to PD. Culturing PD patient‐specific dopaminergic neurons within a Matrigel‐loaded commercial microfluidic platform, for example, *Bolognin* et al.^[^ ^27^ ^]^ demonstrated robust endophenotypes upon comparison to 2D cultures. *Pediaditakis* et al.,^[^ ^18^ ^]^ on the other hand, replicated vascular dysfunction in the context of PD within a membrane‐based platform. The authors spatially separated iPSC‐derived microvascular endothelial cells, seeded within the bottom of the device to form a singular lumen, from iPSC‐derived dopaminergic neurons co‐cultured with primary human astrocytes, pericytes, and microglia within the top compartment of the chamber (Figure 1h).

Pathological phenotypes were induced by the exogenous addition of fibrillar α‐syn, resulting in the formation of phosphorylated α‐syn, mitochondrial impairment, neuroinflammation, as well as compromised barrier function. *Spitz* et al.^[^ ^17^ ^]^ reported a sensor‐integrated microfluidic platform for the long‐term dynamic culture of human midbrain organoids. Next to reduced necrotic core formation, the platform enabled non‐invasive monitoring of essential physiological parameters, including respiratory and electrophysiological activity, as well as dopamine release. Key pathological phenotypes were observed upon introducing patient‐specific midbrain organoids carrying a triplication mutation of the α‐syn gene, including dopaminergic neurodegeneration, α‐syn aggregation as well as mitochondrial dysfunction after 65 days of differentiation. Notably, significant rescue effects were reported after treatment with the repurposed excipient 2‐hydroxypropyl β‐cyclodextrin. A recent publication by *de Rus Jacquet* et al.^[^ ^28^ ^]^ employed the Mimetas OrganoPlate to investigate PD‐associated astrocyte dysfunction. The authors reported that astrocytes from female donors harboring the familial PD mutation LRRK2 G2019S are pro‐inflammatory and fail to support the formation of a functional capillary in vitro. Inhibition of MEK1/2 signaling was shown to disrupt the astrocytic inflammatory profile and rescue BBB formation, implicating its role in PD‐associated dysfunction.

*Osaki* et al.^[^ ^29^ ^]^ reported the first OoC platform for modeling pathological phenotypes of ALS in vitro. Using a compartmentalized device, the authors interconnected iPSC‐derived and light‐sensitive channelrhodopsin‐2–inducible motor neuron spheroids to an engineered skeletal muscle bundle. Light was utilized to stimulate muscle contraction, which subsequently was assessed via micropillar deflection. Introducing iPSC‐derived motor neuron spheroids from a patient with sporadic ALS, resulted in fewer muscle contractions, motor neuron degeneration, as well as increased apoptosis of skeletal myoblasts. Notably, a significant rescue in muscle contraction was observed upon co‐treatment with rapamycin and bosutinib, highlighting the potential of the platform for drug screening studies. *Machado* et al.^[^ ^30^ ^]^ developed a microfluidic neuromuscular junction model to examine the correlation between the familial ALS mutation superoxide dismutase (SOD) 1^G93A^ in astrocytes and ALS dysfunction. Co‐culturing of motor neurons with SOD1^G93A^ glial cells resulted in denervation and reduced myofiber contraction. Pathological phenotypes were rescued upon treatment with the serine/threonine kinase inhibitor 1 necrostatin. A limitation of the study, however, lies in the utilization of mixed animal cell sources, necessitating further investigations using human or patient‐derived cells for enhanced clinical relevance.

As of now the emulation of HD‐associated phenotypes is mostly restricted to 2D applications.^[^ ^6^ ^]^ Employing a membrane‐based microfluidic neurovascular model, *Vatine* et al.,^[^ ^20^ ^]^ however, demonstrated a significant increase in barrier permeability upon comparing microvascular endothelial cells derived from three healthy patients to those derived from an HD patient carrying a 71 CAG repeat in the huntingtin (HTT) gene.

To summarize, the field of modeling NDDs in vitro employing MPS has significantly progressed over the last years, encompassing the replication of critical pathological phenotypes, the use of patient‐specific cell sources, the testing of drugs as well as the integration of non‐invasive sensing strategies. However, current OoC‐based NDD models still largely fail to account for essential factors in brain (patho‐)physiology, including the consideration of the brain's fluid organization system, the role of immune cells, the faithful representation of the native matrix, as well as the emulation of local NDD‐associated microenvironmental changes. Moreover, key aspects such as the need for standardization, as well as the balance of complexity and model robustness often remain ignored in the initial conceptualization of the OoC model, hampering potential clinical applications. Consequently, advancing microphysiological models of the brain beyond the current state and into a clinical setting will necessitate careful consideration of these aspects from the outset. In the remainder of this review, we introduce and discuss five key considerations and criteria (Figure 1) currently largely overlooked in the microphysiological modeling of the human brain and offer a more holistic perspective on emulating NDDs in vitro.

## 2 Fluid Transport in the Brain

**Figure 2:**
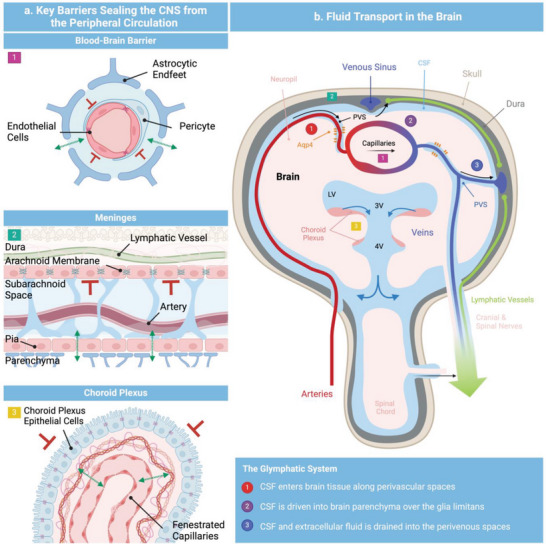
Schematic representation of the various barriers separating the CNS from the peripheral tissues (left panel). Permeable membranes are indicated by green arrows, and tight barriers are illustrated by red perpendicular symbols. Schematic representation of the CSF transport within the brain (left panel). CSF is produced by the choroid plexus within the ventricles entering into the subarachnoid space from where it can enter the glymphatic system or exit into the peripheral circulation. CSF egress routes include meningeal lymphatics, perineuronal pathways, parasagittal spaces, arachnoid villi/ granulations, and adventitia of large cerebral vessels. Schematic representation of the various barriers separating the CNS from the peripheral tissues (right panel). Permeable membranes are indicated by green arrows, and tight barriers are illustrated by red perpendicular symbols.

The groundbreaking identification of the glymphatic system in 2011, its pivotal role in aβ clearance, and the subsequent rediscovery of the meningeal/dural lymphatics in 2015 have led to a notable shift in focus toward the intricate fluid compartmentalization and transport system of the brain in both health and disease in recent years.^[^ ^31^, ^32^ ^]^ This renewed attention highlights the essential role of cerebral homeostasis, governed by a complex interplay between precisely regulated barriers and a unique brain interstitial fluid and cerebral spinal fluid (CSF) clearance mechanism, referred to as the glymphatic system.^[^ ^33^, ^34^, ^35^, ^36^, ^37^ ^]^ To ensure cerebral equilibrium, the CNS is safeguarded from peripheral fluid exchange by three distinct barriers: the BBB at the capillaries i), the arachnoid membrane located in the meninges ii), and the blood‐CSF barrier situated at the choroid plexus iii).^[^ ^33^ ^]^ Comprised of astrocytes, pericytes, and microvascular endothelial cells the BBB constitutes a highly restrictive barrier to transport into the CNS (**Figure** **2**a‐1). The polarized microvascular endothelial cells that make up the tight transcellular and paracellular barrier delineate luminal and abluminal compartments. The latter contributes to the stringent control of the barrier, which is achieved through regulated cellular transport mechanisms that ensure the minimal entry of plasma ultrafiltrate into the neuropil.^[^ ^38^, ^39^ ^]^ As illustrated in Figure 2a‐2, the second layer of protection is conferred by the three‐layered meninges comprised of the dura mater, the arachnoid mater, and the pia mater. The dura mater covers the inner surface of the skull as well as the intervertebral space, surrounding the spinal cord, forming a continuous sheath that envelopes the dural venous sinuses, the middle meningeal arteries as well as the meningeal lymphatic vessels.^[^ ^33^, ^40^ ^]^ Predominantly composed of collagen fibers, the vascularized membrane serves as an immunological interface harboring, among others, neutrophils, *B*‐cells, and *T*‐cells (see Section 5).^[^ ^41^ ^]^ Positioned anatomically below the dura mater, the arachnoid membrane effectively restricts the passage of solutes and fluid between the CNS and the peripheral tissues. Low barrier permeability is attained by leptomeningeal fibroblasts and tight junctions. The arachnoid mater and the pia mater are connected via the so‐called subarachnoid space, the main location of CSF next to the ventricular system. In contrast to the arachnoid mater, the pia mater, a monolayer of cells loosely adhering to the brain and spinal cord does not provide a barrier to CSF influx.^[^ ^33^, ^40^ ^]^

**Figure 3:**
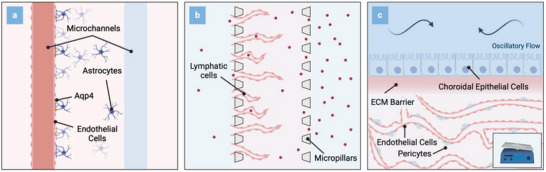
Schematic representations (top view) of three potentially applicable microfluidic setups to study fluid transport in the human brain in vitro: a) Microfluidic platform comprised of two microchannels embedded in an astrocyte‐laden hydrogel to study glymphatic influx. b) Compartmentalized platform to investigate lymphatic clearance. c) Microfluidic device interconnecting vascular networks with a choroidal barrier to emulate the choroid plexus. Oscillatory flow patterns induce physiological barrier properties.

While this controlled interplay of CNS barriers safeguards the brain, the concomitant lack of lymphatic drainage within the neuropil harbor a risk of stasis and, thus, accumulation of toxic protein waste such as, e.g., aβ or α‐syn.^[^ ^31^, ^42^ ^]^ To counteract the latter, the brain employs an alternative clearance mechanism that acts as a functional substitute for transcapillary fluid in‐/efflux.^[^ ^35^ ^]^ This mechanism utilizes CSF, an ultrafiltrate of plasma predominantly produced within the ventricular system of the brain (see Figure 2b). The ventricular system, built up by four ventricles each holding 25 mL of CSF, is lined by the choroid plexus, a highly vascularized secretory epithelium with papillary‐like extensions projecting into the lumen of the ventricles.^[^ ^43^ ^]^ The epithelium reinforced by tight junctions forms the third selective CNS barrier separating the CSF from the peripheral blood circulation. The influx of an ultrafiltrate of plasma toward the choroidal membrane originates from a dense reticular network of fenestrated capillaries, as depicted in Figure 2a‐3. From the ventricles, the CSF drains into the basal cisterns, while a minor portion flows down in the conus medullaris of the spinal cord.^[^ ^33^, ^44^ ^]^

The influx of CSF into the neuropil initiates at the basal cistern. Here, CSF enters and moves along the perivascular space, a network of passageways located around arterioles, capillaries, and venules (Figure 2b).^[^ ^45^ ^]^ CSF travels along the perivascular space until it reaches the vascular segment, where the pial basement membrane disappears and the vascular and glial basement membranes fuse.^[^ ^33^ ^]^ Subsequently, the CSF passes beyond the glial basement membrane either through the clefts of astrocytic endfeet or in a process involving aquaporin‐4 (Aqp4), a water channel accounting for up to 60% of the endfeet surface area.^[^ ^46^ ^]^

Upon entering the parenchyma, the CSF mixes with the residing interstitial fluid before moving along the extracellular space, which constitutes 14–24% of the brain's volume fraction.^[^ ^33^, ^47^ ^]^ Due to the high – albeit variable – hydraulic resistance observed in the neuropil, the exact mechanisms of intraparenchymal fluid flow remain to be discovered.^[^ ^33^ ^]^ Tracers of various size (900–69,000 Da), however, have been shown to be cleared at similar rates (0.11 µL g brain^−1^ min^−1^) despite an up to fivefold difference in diffusion coefficient, pointing toward fluid advection.^[^ ^48^ ^]^ As of now, three potential transport mechanisms along the brain parenchyma have been postulated: 1) CSF continues along the perivascular space of the capillaries where it ultimately enters that of the venules, 2) the entirety of the CSF moves through the brain parenchyma from where it egresses into the venous perivascular space and 3) CSF moves along both aforementioned pathways or via a yet unknown mechanism.^[^ ^33^ ^]^

### 2.1 CSF Egress Routes

1. *Perineural Egress* serves as a significant route for CSF efflux.^[^ ^33^, ^49^ ^]^ Tracers delivered to the subarachnoid space are detected in the epineurium and within the endoneurial fluid of peripheral nerves. This route is conserved across different species, with nearly all cranial nerves contributing to CSF egress. Among them, the olfactory nerves constitute the largest outflow path.
2. *Dural Lymphatics* located in close proximity to the large vessels of the dura mater, exhibit uptake of intrathecally delivered tracers. While earlier studies emphasized the main uptake at basal dural lymphatics, recent research highlights the significant role of dorsal lymphatics in CSF egress.
3. *Arachnoid Villi/Granulations* denote invaginations of the arachnoid membrane. Drainage through these structures has been suggested to occur along transcellular and paracellular pathways. Transcellular pathways involve transcytosis, while paracellular pathways are believed to egress through extracellular cisternal spaces. Sparse granulation in rodents and human children suggests this egress pathway to be less substantial than others.
4. *Dural Spaces along Venous Sinuses* reveal Gadobutrol accumulation, a nonionic hydrophilic compound impermeable to the BBB. Final egress pathways from the dural parasagittal space are still unknown.
5. *Adventitia of Major Cerebral Vessels* shows tracer accumulation upon intracisternal administration. Egress along adventitia has not been extensively studied yet.
6. *Spinal Cord Egress* has been reported for cranial CSF. Draining around nerve roots may be facilitated by dural lymphatics and arachnoid granulations located close to nerve roots.

The final step of the clearance cascade is the removal of CSF from the CNS into the peripheral circulation. As any excess of CSF within the brain would result in a significant increase in intracranial pressure, CSF efflux closely matches its de novo formation of ≈600 mL/day.^[^ ^33^ ^]^ While the specific contributions of the individual egress routes (Section 2.1), such as the dural lymphatics or the arachnoid villi to CSF removal remain unclear, the olfactory pathway has been established as a primary course of CSF egress.^[^ ^32^, ^49^, ^50^, ^51^, ^52^, ^53^, ^54^, ^55^, ^56^, ^57^, ^58^ ^]^ Recognizing the crucial role of the brain's fluid transport system in facilitating brain clearance, any deficiencies therein can adversely affect brain homeostasis. This is underscored by the observation of aberrations within the brain's clearance system in the pathology of many neurological disorders including NDDs. While pathological phenotypes vary among individual disorders, common observations include impaired glymphatic clearance, Aqp4 depolarization as well as reduced CSF drainage.^[^ ^59^, ^60^, ^61^, ^62^ ^]^ **Table** **1** provides a comprehensive overview of key pathological changes within the brain fluid transport system observed in NDDs and corresponding models.

**Table 1:** Alterations in the Brain Fluid Transport System in NDDs.

NDD	Clearance	Aqp4	CSF drainage	Lymphatic Clearance	Reference
AD	AQP4 K/O: ↓ Tau clearance AQP4 K/O in APP/PS1 mice: 25–50% ↑ in soluble and insoluble aβ ↑ intraneuronal aβ aggregates APP/PS1mice: Glymphatic clearance ↓ precedes aβ deposition	Depolarization reported in AD patients and AD models	AD patients: 33% ↓ in the CSF clearance rate ↓ CSF drainage along the olfactory nerves abnormal olfactory nerve morphology	Meningeal lymphatics K/O: ↓ aβ No pathological changes to dural lymphatics were reported by amyloidosis Ablation of dorsal dural lymphatics or ligation of deep cervical lymph nodes: ↑ aβ and Tau in meninges and parenchyma	[33, 63, 64, 65, 66, 67, 68, 69, 70]
PD	AQP4 K/O: ↓ α‐syn clearance after intrastriatal injection PD patients: lower diffusivity in PVS	A53T mice: Loss of Aqp4 polarization ↑ AQP4 accumulation in proximity to α‐syn positive neurons PD patients: ↑ AQP4 immunoreactivity Inverse relationship between AQP4 expression and α‐syn levels	PD patients: CSF drainage toward dural lymphatics and into the cervical lymphatic system perturbed	PD patients: no distinct morphological differences in lymphatic vasculature In vivo PD models: ↓ in occludin and ZO‐1 in dural lymphatic vasculature A53T model: cervical lymphatic vessel ligation ↑ α‐syn aggregation	[42, 71, 72, 73]
ALS	AQP4 K/O: ↑ toxic variants of superoxide dismutase‐1 In vivo models: Impaired clearance	ALS in vivo models: Depolarization ↑ GFAP ↑ AQP4 ALS patients: No depolarization ↑ AQP4			[33, 74, 75]
HD	Extracellular huntingtin transport facilitated by the glymphatic system				[76]

### 2.2 Modeling the CNS Fluid Organization System in MPS

The key role of the fluid organization system in maintaining brain homeostasis and its resulting implications necessitates its consideration in the design of future MPS. Significant progress has been made in recent years in modeling the BBB using MPS, reflecting its pivotal role in CNS drug delivery.^[^ ^13^, ^77^ ^]^ This progress encompasses a spectrum of models from barrier‐based^[^ ^18^ ^]^ and sensor‐integrated setups^[^ ^78^, ^79^ ^]^ up to perfusable self‐assembled microvascular networks^[^ ^15^ ^]^ (**Figure** **3**). We refrain here from an exhaustive exploration of individual MPS of the BBB and refer the readers to an in‐depth review by *Hajal* et al.^[^ ^13^ ^]^ With few exceptions, research on other compartments of the fluid transport system beyond the BBB, however, has been limited. One of these exceptions is a recently published study by *Lim* et al.,^[^ ^80^ ^]^ who reported a microphysiological model of the choroid plexus. Using a rocking platform*, Lim* et al. produced a dynamic, reversing flow to assess flow‐related changes within the choroidal tissue. In their model, the authors co‐cultured a monolayer of choroidal epithelial cells in proximity to a self‐assembled capillary network using microvascular endothelial cells and brain vascular pericytes. In addition to a significant reduction in occludin within the capillaries, a closer approximation of the local fenestrated vasculature, and dynamic culture resulted in a significant upregulation of claudin‐1 as well as RSPH9, two markers of the epithelial tissue.

Furthermore, dynamic culture improved the secretory phenotype of the engineered choroidal tissues and markedly increased the cytotoxic impact of trastuzumab upon introducing macrophages and breast cancer cells into the system. The platform, thus, might serve as a promising tool for evaluating pathological alterations at the choroidal interface during neurological disorders, such as NDDs, including local immune cell contributions. Another noteworthy study was published by *Soden* et al.,^[^ ^81^ ^]^ who reported a simplified model of the glymphatic system. To emulate the glymphatic influx in vitro, the authors developed a platform with two parallel endothelial cell‐seeded microchannels within an astrocyte‐seeded hydrogel compartment. Next to the replication of Aqp4 polarization at the astrocytic endfeet, the microfluidic co‐culture facilitated the investigation of fluid conduction along the parenchymal interface. The results demonstrated a notable reduction in volume drainage under conditions of lipopolysaccharide‐mediated inflammation, treatment with aβ_42_, and Aqp4 inhibition using TGN‐20. In addition to mechanistic investigations, the platform could be utilized to explore patient‐specific differences in the context of NDDs by introducing iPSC‐derived cells. *Serrano* et al.^[^ ^82^ ^]^ developed a microfluidic model that harnesses the self‐assembling properties of lymphatic endothelial cells coupled with the capability of MPS to apply controlled flow regimes to generate lymphatic capillaries in vitro. The model replicated physiological drainage rates of interstitial proteins and molecules and was further utilized to simulate the recruitment of immune cells toward the lymphatic system making it a promising tool for the in vitro exploration of the meningeal lymphatics.

Acknowledging the complex interplay amongst the various flow pathways as well as persistent uncertainties in underlying mechanisms, e.g. glymphatic flow profiles, a comprehensive emulation in MPS currently remains out of reach. However, MPS may provide a powerful tool to mimic many of the distinct subunits or mechanisms of fluid transport in vitro. This is of considerable importance in the context of NDDs where the unique architecture and organization of the brain clearance system might play a pivotal role. Furthermore, increased knowledge of the brain fluid transport system can be exploited for improved drug delivery, thereby presenting a promising pathway for advancing targeted therapeutic strategies not only for NDDs but also for brain cancers and other neurological diseases. With an increase in the complexity of MPS, a more holistic approach has to be pursued in the initial platform designs, which, among others, can include the development of modular systems or the integration of computational modeling (see Section 6). For a comprehensive review of brain fluid transport, please refer to *Rasmussen* et al.^[^ ^33^ ^]^ For more information on how the glymphatic system can be emulated using MPS see *Spitz* et al.^[^ ^83^ ^]^

## 3 Immune‐Competent Models of the Brain

The innate immune system serves as the body's initial defense mechanism against infections.^[^ ^84^ ^]^ In recent years, the activation of the innate immune system has been recognized as a critical factor in neurodegeneration.^[^ ^85^ ^]^ Inflammatory mediators released upon immune cell activation can compromise the function and structure of surrounding neurons, driving the pathogenesis of NDDs. Given the implication of chronic inflammation in the early pathogenesis of NDDs, a comprehensive investigation of the innate immune system's involvement and the intricacies of its mechanisms becomes indispensable. Such investigations are pivotal for advancing diagnostic modalities and therapeutic interventions in NDDs.^[^ ^85^ ^]^

**Figure 4:**
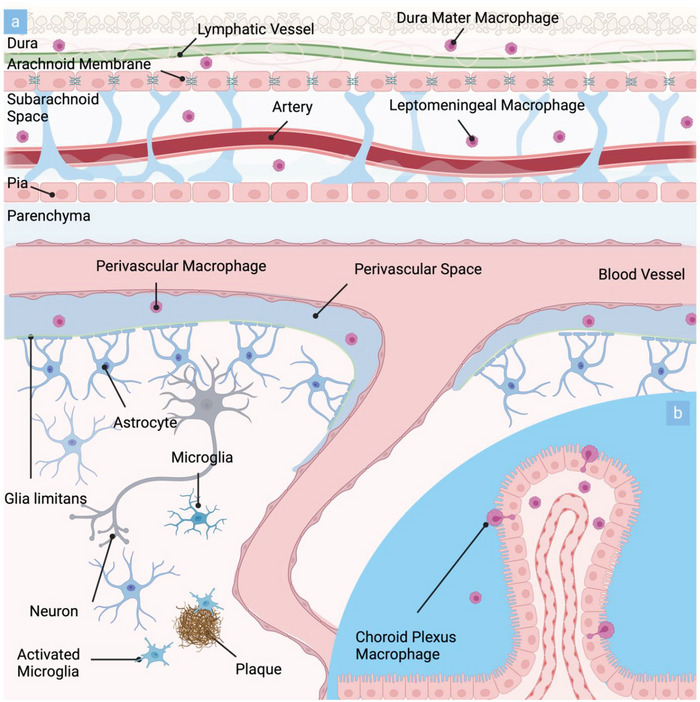
Schematic representation of the brain's innate immune system comprised of CAMs and microglia and their respective locations in vivo: a) Cross‐sectional representation of the human brain highlighting the parenchymal location of microglia and their activation in proximity to aβ plaques. CAMs are located in the dura mater, the leptomeninges as well as the perivascular space. b) Inset demonstrates the location of CAMs within the choroid plexus of the brain.

The brain's innate immune system is comprised of highly specialized tissue‐resident macrophages distributed throughout various regions, constituting ≈10% of all CNS cells.^[^ ^86^ ^]^ While microglia are restricted to the brain parenchyma, CNS‐associated macrophages (CAMs) are found at CNS interfaces including the meninges (leptomeningeal macrophages), the perivascular space, and the choroid plexus (see **Figure** **4**).

### 3.1 Microglia and NDDs

Microglia are the predominant component of the brain's innate immune system. Derived from erythromyeloid progenitors located in the mesodermal yolk sac, microglia migrate into the CNS during early embryonic development.^[^ ^87^, ^88^ ^]^ The resident immune cell performs different functions depending on the stage of ontogenesis, encompassing the release of diffusible factors as well as the phagocytosis of synaptic elements, cellular debris, living cells, and axons. During the embryonic stage, microglia promote vasculogenesis and assist in synapse formation through the secretion of hormones,^[^ ^89^, ^90^ ^]^ while in postnatal development they promote neurogenesis and shape neuronal circuits by eliminating apoptotic neurons through phagocytosis.^[^ ^91^ ^]^ In subsequent stages of life, microglia are responsible for maintaining tissue homeostasis, remodeling, and sustaining the extracellular matrix (ECM), as well as regulating neuronal integrity through synaptic remodeling and secretion of neurotrophic factors.^[^ ^92^, ^93^, ^94^, ^95^, ^96^ ^]^ However, in the case of NDDs, loss, and perturbations of physiological microglial function can occur, contributing to disease progression. The microglial cell membrane is equipped with innate immune receptors, such as pattern recognition and toll‐like receptors, enabling the response to pathogen‐associated and danger‐associated molecular patterns, as well as many endogenous proteins involved in NDDs such as aβ, α‐syn, mutant HTT (mHTT), mutant SOD1, and the interleukin‐1β (IL‐1β) family.^[^ ^97^, ^98^, ^99^, ^100^ ^]^ In several NDDs, microglia are exposed to high levels of immune activators, triggering microglial activation. Depending on the signaling strength, the involved region of the brain, and the environmental conditions, glial activation can have multiple consequences ranging from enhanced clearance of debris and resolution of inflammation to the secretion of a wide range of inflammatory mediators such as ROS, nitric oxide (NO), myeloperoxidase, inducible NO synthase, and ECM mediators.^[^ ^85^, ^96^, ^101^, ^102^ ^]^ Although the innate immune system is intended to protect the brain, excessive microglial reaction and sustained release of pro‐inflammatory mediators as seen in NDDs, can be detrimental, further promoting disease progression. Microglia‐driven neuroinflammation has been shown to be involved in the suppression of axonal transport and adult neurogenesis, impaired synaptic plasticity, and reduced supply of neurotrophic factors to glial cells causing neuronal dysfunction and loss.^[^ ^103^, ^104^, ^105^, ^106^, ^107^ ^]^ Further mechanisms of microglial activation in response to different NDDs can be found in **Table** **2**.

**Table 2:** Role of innate immune cell activation in the pathogenesis of AD, PD, ALS, and HD.

NDD	Microglia	Reference	CNS‐associated macrophages	Reference
AD	Immune response initiators:aβ, tauMicroglia activation response: ↑ TNF‐α ↑ IL‐1β ↑ NF‐kβ signaling ↑ IL‐12 subunit‐β ↑ NOS	[85, 149, 150, 151, 152, 153]	Immune response initiators:aβ, tauCAMs activation response: ↑ vesicle accumulation ↑ ROS ↑ NADPH oxidase 2 ↑ IL‐6 ↑ TNF‐α ↑ IL‐1β	[97, 112, 154, 155, 156]
PD	Immune response initiators:α‐synMicroglia activation response: ↑ IL‐1β ↑ TLR ↑ TNF‐α ↑ IL‐6 ↑ IFN‐gamma ↑ NRS	[106, 157, 158, 159, 160, 161, 162]	Immune response initiators:α‐synCAMs activation response: ↑ uptake of α‐syn ↑ pro‐inflammatory cytokines	[163]
HD, ALS	Immune response initiators:HTT, TDP43, tau, SOD1Microglia activation response: ↑ Pro‐inflammatory cytokines ↑ ROS ↑ NF‐kβ signaling ↑ IL‐1b ↑ CCL2, CCL4, CCl11, CCL26 ↑ IL6 ↑ IL8 ↑ TNF‐α ↓ neuronal activity ↑ plasma levels of clusterin	[164, 165, 166, 167, 168, 169, 170, 171]	Immune response initiators:HTTCAMs activation response: ↑ IL‐1β ↑ IL6 ↑ IL8 ↑ IL10 ↑ IL12 ↑ TNF‐α ↑ p70	[172]

### 3.2 CNS‐Associated Macrophages and NDDs

In Section 2, we highlighted how the human CNS is shielded from external influences by distinctive anatomical structures. These structures act as specialized barriers and interfaces, intricately controlling the flow of circulating immune cells, immune‐active metabolites, and signaling molecules.^[^ ^39^, ^108^ ^]^ Along these interfaces—such as the meninges, the perivascular space, and the choroid plexus—populations of tissue‐resident macrophages are found (refer to Figure 4a), suggesting their involvement in the intricate defense mechanisms of the CNS.^[^ ^109^ ^]^ CAMs encompass perivascular macrophages, choroid plexus macrophages, leptomeningeal as well as dural macrophages.^[^ ^86^, ^110^, ^111^, ^112^, ^113^ ^]^ Similarly to microglia, CAMs are derived from embryonic progenitors in the embryonic yolk sac or embryonic progenitor descendants in the fetal liver.^[^ ^87^, ^114^ ^]^ They can be distinguished by their localization within the CNS interfaces as well as by variations in their morphology.^[^ ^115^ ^]^ While perivascular, leptomeningeal, and dural macrophages are characterized by an elongated morphology, choroid plexus macrophages display a stellate shape.^[^ ^115^, ^116^, ^117^ ^]^ Due to their ability to phagocytose and migrate, CAM subpopulations act as important control checkpoints of CNS gateways, regulating the immune response at CNS borders. Perivascular and leptomeningeal macrophage localization at the interface between the parenchyma and the bloodstream suggests a potential role in supporting barrier function by monitoring CFS drainage and filtering of antigens, metabolites, and other molecules.^[^ ^118^, ^119^ ^]^ Moreover, perivascular macrophages are proposed to moderate BBB permeability and control metabolic processes via regulation of glucose, lipids, and iron uptake.^[^ ^116^, ^120^, ^121^ ^]^ However, the exact functions of individual CAMs are still unclear and very few studies have investigated CAM diversity in healthy or diseased human brains.^[^ ^112^ ^]^ Inflammatory‐associated factors produced by CAMs could initiate modifications in the brain vasculature and cells of the perivascular space.^[^ ^122^ ^]^ The lack of knowledge can be attributed to the low numbers of CAMs within the CNS, compounded by the lack of precise tools capable of selectively targeting and manipulating myeloid cells while discerning them from the prevailing microglia population. As a result, the isolation and investigation of these cells and their respective functions pose significant challenges, impeding progress in unraveling their roles within the CNS.^[^ ^109^ ^]^ A few studies explored the connection between CAMs, neuroinflammation, and NDDs.^[^ ^109^ ^]^ The potential functional diversity of CAMs in NDDs suggests that a comprehensive characterization of their roles during NDDs onset and progression is needed. A summary of CAM activation mechanisms observed in major NDDs can be found in Table 2.

### 3.3 MPS as a Tool to Investigate the Role of the Innate Immune System in NDDs

Inflammation is a natural reaction of the innate immune system in response to stressful stimuli such as infections, harmful deposits of metabolites, and tissue injury. Once normal tissue homeostasis is restored, inflammation is resolved. However, a persisting inflammatory response that leads to chronic effects of the immune stressors could trigger tissue pathologies.^[^ ^123^ ^]^ It is well known that neuroinflammation accompanies NDDs, and numerous findings indicate that in some NDDs, neuroinflammation is both a consequence and a trigger of the pathology.^[^ ^85^, ^123^ ^]^ Therefore, innate immune activation might play a role in the etiology and course of the disease. Increased understanding of these processes is crucial to developing anti‐inflammatory therapies aimed at modulating neuroimmune signaling in the context of NDDs.

While microglial functions have been intensively investigated in animal models, with the majority of research being performed in rodents, relatively little research has been done to assess whether these findings could be translated to humans.^[^ ^124^ ^]^ Several works emphasize the difference between murine and human microglial mechanisms in the immune response and most studies on CAMs are made in non‐human animal models.^[^ ^125^, ^126^, ^127^ ^]^ Therefore, studies to elucidate the ontogeny and function of these cells in humans or human‐based models are urgently needed. One promising tool to study microglial function in vitro is human brain organoids.^[^ ^128^, ^129^, ^130^, ^131^ ^]^ *Sabate‐Soler* et al.,^[^ ^132^ ^]^ for example, reported the successful co‐culture of human midbrain organoids with iPSC‐derived microglia. Next to reducing the number of apoptotic cells and lowering oxidative stress, microglia in midbrain organoids were shown to affect synaptic remodeling and increase neuronal excitability. While multiple studies on microglia‐integrated organoids have been published, limited progress has been made in investigating microglial dysfunction in organoid‐based models of NDDs.^[^ ^133^ ^]^ One notable exception constitutes a publication by *Lin* et al.,^[^ ^134^ ^]^ which demonstrated impaired microglial function including reduced aβ clearance upon co‐culturing cerebral organoids with microglia carrying an APOE4 mutation. Current microglial protocols differ in cell origin, proportion, and fidelity to the native microglial state, which may produce inconsistent results. For this reason, robust and reproducible methods to recapitulate in vivo signatures are needed.^[^ ^133^ ^]^

To date, only a handful of studies have started to incorporate microglia into OoC platforms. One of the first microfluidic studies aimed at better understanding microglial activation in vitro was published by *Cho* et al.,^[^ ^135^ ^]^ who utilized a microfluidic chemotaxis platform to monitor the response of microglia to aβ gradients over several weeks. *Achyuta* et al.^[^ ^136^ ^]^ demonstrated microglial activation within a compartmentalized microfluidic device, recapitulating the neurovascular unit. TNF‐α stimulation on the vascular side of the modular platform triggered immune cell activation in the parenchymal compartment of the device. In NDDs, microglia activation induces neurotoxic phenotypes in astrocytes, which in turn exhibit immunomodulatory effects by releasing and responding to immune system mediators.^[^ ^137^ ^]^ Despite our increasing understanding of their individual functions, little is known about the crosstalk between microglia and astrocytes.^[^ ^138^ ^]^ One platform that could be employed to further investigate the intricate glial relationship constitutes a device reported by *Park* et al.^[^ ^26^ ^]^ As previously described the study reported a 3D human tri‐culture model capable of replicating critical AD‐associated phenotypes in vitro. Microglia recruitment and activation, as well as toxicity to neurons and astrocytes, were reproduced by introducing microglia at different stages of phenotype progression. In a follow‐up study, the group demonstrated an increase in microglial activation upon peripheral immune cell infiltration, supporting the importance of a system that can recapitulate both complex microenvironments and cellular interactions.^[^ ^139^ ^]^ *Trapecar* et al.,^[^ ^140^ ^]^ developed a polycarbonate‐based mesofluidic platform to study gut‐liver‐cerebral interactions in the context of PD. Multi‐tissue culture improved microglia maturation within the PD compartment, comprising patient‐derived neurons, astrocytes, and microglia, while circulating *T*‐cells elicited microglial activation. The work underlines the importance of systemic interactions in achieving more in vivo‐like phenotypes of the brain.

As of now, only a handful of studies have started to incorporate cells of the innate immune system. Several aspects need to be considered to more closely mimic CNS–immune interactions in future MPS models including i) microglial and CAM functions are not interchangeable and their role and density distribution in different tissues is driven by the tissue microenvironment,^[^ ^84^, ^141^, ^142^, ^143^, ^144^, ^145^ ^]^ ii) multi‐cellular interactions and tissue architecture affect inflammatory pathways in microglia response,^[^ ^146^ ^]^ iii) variations in microglia cell density and distribution may alter the innate immune response dynamics and potentially play a role in NDD predisposition.^[^ ^85^ ^]^

Advanced single‐cell analysis led to the discovery of novel microglia clusters, termed disease‐associated microglia in AD and ALS. In the future, we need to understand the role of microglia subsets in the onset and progression of NDDs. Possible therapeutic interventions that could be tested in OoC platforms include drugs targeting genes of disease‐associated microglia.^[^ ^147^, ^148^ ^]^ Lastly, our current understanding of the precise role of human CAMs in NDDs remains limited. Protocols for the differentiation of CAMs from iPSCs will help to elucidate the immune cells’ (patho)physiology, ontogeny, and function. To summarize, incorporating the innate immune cells of the CNS in future MPS will allow researchers to unravel the genetic and environmental mechanisms that drive neurodegeneration in humans. These in vitro systems will become pivotal for the development of diagnostic tools, pharmaceutical treatments, and ultimately, for finding ways to guide the immune system toward regeneration.

## 4 Recapitulating the Native ECM of the Brain

Another critical factor often overlooked in the simulation of neurological tissues in vitro is the faithful replication of the native tissue's ECM. The brain's ECM, aside from its mechanical properties, plays a pivotal role in shaping key cellular processes such as proliferation, differentiation, migration, as well as functional maturation (e.g., axon myelination, synaptogenesis).^[^ ^173^ ^]^ Consequently, a closer consideration of the native ECM becomes indispensable as microphysiological models of the brain progress.

**Figure 5:**
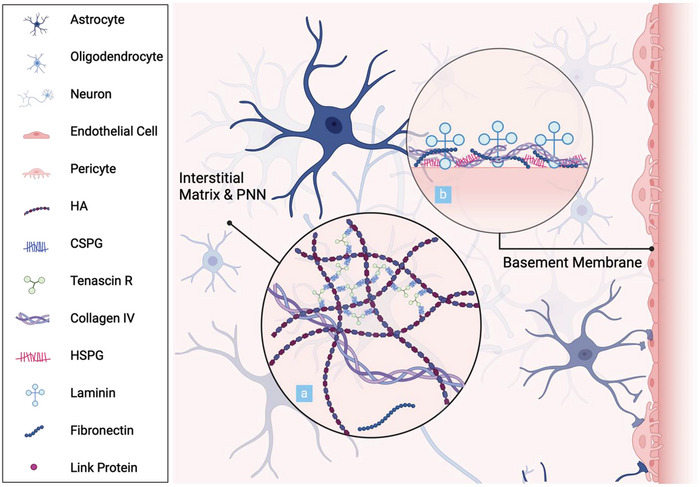
Graphical illustration of the cell and matrix composition of the brain parenchyma: a) Graphical representation of the interstitial matrix and the ternary complexes of the perineuronal nets. b) Schematic depiction of the structural arrangement of the basement membrane.

The ECM of the CNS, distinct from peripheral tissues, forms a structured lattice of amorphous aggregates with a low proportion of fibrous proteins (e.g., fibronectin and collagen).^[^ ^174^ ^]^ Constituting ≈20% of the native tissue, the brain's ECM primarily consists of the glycosaminoglycan hyaluronic acid (HA), chondroitin sulfate proteoglycans (CSPG), heparan sulfate proteoglycans (HPSG), link proteins, and glycoproteins such as tenascins, laminins, and reelin.^[^ ^175^, ^176^ ^]^ The composition of the ECM varies by location, with three distinct subtypes: i) the basement membrane, ii) the interstitial matrix, and iii) the perineuronal net.^[^ ^177^ ^]^ Illustrated in **Figure** **5**, the basement membrane comprises sheet‐like ECM layers enveloping the brain vasculature and surrounding the pial surface. The constituents of the basement membrane, while variable, encompass collagen IV, laminins, nidogens, fibronectin, and HSPGs. Beyond serving as a major fluid pathway, the basement membrane plays a pivotal role in brain development and in the maintenance of the BBB.^[^ ^33^, ^178^ ^]^ The interstitial matrix, composed of HA, proteoglycans, tenascins, link proteins, and glycoproteins, constitutes the highest fraction of the brain's ECM. Apart from its highly negative charge, growth factors, and neuromodulatory agents, it plays a critical role in maintaining optimal hydration capacity—a prerequisite for sustaining physiological brain activity.^[^ ^179^ ^]^

Perineuronal nets, specialized structures within the interstitial matrix, consist of ternary complexes of CSPGs, tenascin glycoproteins, and link proteins, connected to an HA backbone. This mesh‐like matrix organizes into distinct assemblies surrounding the synapses of select neuronal subpopulations, where it contributes to synaptic stabilization, neuroprotection, and ionic buffering.^[^ ^180^ ^]^

The native ECM exhibits notable plasticity, undergoing constant dynamic change throughout processes such as neurodevelopment, learning, as well as aging.^[^ ^173^, ^181^, ^182^ ^]^ This malleability is mediated by endogenous proteases, including metalloproteinases, cathepsins, and plasminogen activators.^[^ ^181^ ^]^ Given the importance of the ECM under physiological conditions, pathological alterations raise concerns for significant repercussions. While it remains unclear whether changes in the ECM precede or follow neuronal dysfunction, studies have reported the former in several neurological disorders, including NDDs.^[^ ^174^, ^178^, ^183^, ^184^ ^]^ As summarized in **Table** **3**, modifications in NDDs frequently coincide with protein aggregations or lesions, prompting inquiry into their potential role in protein aggregation. Next to compositional changes of the ECM, studies have reported alterations in the mechanical properties of the neurodegenerative brain. Magnetic resonance elastography measurements, for example, have shown significant reductions in brain stiffness in AD patients at the macroscale upon comparison to healthy controls.^[^ ^185^ ^]^ On the nanoscale, Young's moduli of aβ‐fibrils, on the other hand, were reported in the GPa range, indicating a notable contrast in the mechanical properties between plaques and the surrounding brain tissues.^[^ ^182^, ^186^ ^]^ Additionally, a significant decrease in viscoelasticity has been reported in the NDDs AD, PD, and MS, highlighting the comprehensive impact of these pathologies on the brain's ECM.^[^ ^182^, ^187^, ^188^ ^]^

**Table 3:** Spatio‐temporal changes in the human brain's ECM in NDDs.

Brain ECM	Constituents	NDD	Spatio‐Temporal Dynamics in NDDs	Reference
Interstitial Matrix & PNN	HA	AD	↑ at the perimeter of plaques	[189]
		MS	↑ High‐MW HA accumulation in chronic lesions ↑ Low‐MW HA in acute lesions	[183, 190]
	Proteoglycans	AD	CSPGs present in neurofibrillary tangles and senile plaquesDSPGs associated with senile plaquesKSPGs located at synapses and neurites within plaquesDecorin associated with periphery of plaques and neurofibrillary tanglesHSPG Agrin ↑ around senile plaques and hyperphosphorylated tau Around senile plaques and neurofibrillary tangles glypican and syndecan ↑	[174, 191, 192, 193]
		PD	HSPG Accumulation of agrin in Lewy body Versican expression ↑	[194, 195]
		MS	Aggrecan, versican, neurocan, dermatan sulfate proteoglycans ↑ around lesions Aggrecan, versican, neurocan, dermatan sulfate proteoglycans ↓ in the center of lesions	[196]
	Glycoproteins	AD	Reelin ↓ Tenascin C associated with cored plaques Tenascin C & R significantly ↑ in CSF of female patients Thrombospondin‐1 ↓	[197, 198, 199, 200]
		MS	Tenascin C & R In MS lesions ↑ Around lesions ↓ Laminin ↑	[174, 201]
Basement Membrane	Collagen	AD	Collagen IV ↑ Collagen IV ↓ in AD neuropathological change	[178, 184, 202]
		ALS	Collagen IV ↑	[203]
		PD	Collagen I expression ↑ Collagen IV ↑	[195, 204]
	Fibronectin	AD	↑	[184, 205]
	Proteoglycans	AD	HSPGs ↑ Perlecan ↑	[184, 206]
	Glycoproteins	AD	Laminin Fragmentation ↑ ↓ in AD neuropathological change Nidogen ↓Agrin ↑	[184, 202, 205, 207, 208, 209, 210, 211]
		ALS	Laminin ↑	[212, 213]

To conclude, a thorough understanding of the native tissues’ composition and architecture is crucial to designing biomaterials that intentionally engage with cells and effectively replicate (patho)physiological microenvironments in vitro. Given their close approximation to the native tissue, their applicability for additive manufacturing strategies, as well as their suitability for MPS, the following section will exclusively focus on hydrogel‐based biomaterials.

### 4.1 Natural and Engineered/Synthetic Hydrogels for Neurovascular Modeling

Microphysiological models of the neurovascular unit or its constituents predominantly rely on the use of naturally derived hydrogels, with Matrigel, fibrin, and collagen constituting the most commonly employed matrices. Matrigel, a murine sarcoma‐derived basement membrane comprised of laminin (≈60%), collagen IV (≈30%), entactin (≈8%), and perlecan (≈2–3%) presents the most widely used matrix in neural tissue engineering.^[^ ^214^ ^]^ However, in addition to challenges posed by high batch‐to‐batch variability, animal origin, and sourcing, Matrigel exhibits limited resemblance to the interstitial ECM, as evidenced by the lack of key glycoproteins and proteoglycans.^[^ ^215^ ^]^ Consequently, recent attention has shifted toward the development of matrices with enhanced biomimicry, with decellularized brain ECM emerging as a promising alternative. *Simsa* et al.,^[^ ^216^ ^]^ for example, have demonstrated the applicability of decellularized porcine brain ECM hydrogels for cerebral organoid differentiation. Their study reported similar gene expression profiles upon comparing the reconstituted matrix to Matrigel‐derived controls. To enhance the biomimicry of Matrigel in a microfluidic platform for cerebral organoid differentiation, *Cho* et al.^[^ ^14^ ^]^ supplemented the biomatrix with human decellularized brain ECM, resulting in enhanced neurogenesis, increased neuronal and radial glial populations, and improved cortical layer development. No significant changes were observed upon comparing human and porcine‐derived brain ECM, the latter providing a more accessible alternative. Notably, decellularized brain ECM also showed promise in additive manufacturing. *Yi* et al.^[^ ^217^ ^]^ bioprinted patient‐derived tumor cells, vascular endothelial cells, and decellularized ECM into a cancer–stroma concentric‐ring structure, capable of replicating important characteristics of the in vivo tumor microenvironment (e.g., radial oxygen gradient). While different cellular responses have been observed in reaction to decellularized ECM from distinct regions of the brain, it remains unclear if decellularized brain ECM‐based hydrogels favor the formation of a tight vascular interface.^[^ ^218^ ^]^ From a neurovascular perspective, the two most utilized natural matrices are fibrin and collagen. After seven days of culture, self‐assembled vascular networks, a prominent application of fibrin hydrogels, have been shown to locally deposit constituents of the native ECM, including collagen IV, laminin, and HSPG2.^[^ ^219^ ^]^ Given its origin, however, next to increased susceptibility to protease‐mediated degradation, fibrin fails to fully mimic the neurovascular environment.^[^ ^215^ ^]^ Moreover, the neurotoxic properties of thrombin, utilized in the fibrinogen‐to‐fibrin conversion, render fibrin hydrogels unfavorable for neurovascular cocultures. To address this limitation, *Ko* et al.^[^ ^25^ ^]^ recently developed a coculture platform with Matrigel‐encapsulated neuronal tissues in one compartment surrounded by a fibrin‐based vascular bed in a second compartment, allowing the generation of perfusable networks adjacent to Matrigel‐embedded AD‐neurospheres. Alternatively, collagen hydrogels have been utilized in microphysiological models of the neurovascular unit. For instance, *Herland* et al.^[^ ^220^ ^]^ reported a 3D model of the human BBB by seeding microvascular endothelial cells into a single hollow lumen within a collagen I hydrogel enriched with astrocytes. Similarly, *Osaki* et al.^[^ ^221^ ^]^ demonstrated the successful coculture of neurospheres with perfusable vascular networks in a collagen hydrogel under interstitial fluid flow conditions. However, next to long polymerization times – a modulator of cell distribution –, batch‐to‐batch and inter‐species variabilities and the inability to faithfully replicate the native ECM, continue to limit the applicability of collagen for advanced models.^[^ ^215^ ^]^ To better emulate the native basement membrane matrix within their neurovascular PD model, *Pediaditakis* et al.^[^ ^18^ ^]^ coated their platform with a combination of collagen IV, laminin, and fibronectin. While native ECM constituents have been employed in in vitro cultures, their inability to form hydrogels on their own (e.g., fibronectin, laminin) or their inherent lack of cell binding motifs (e.g., HA), either restricts them to coating approaches (see *Pediaditakis* et al.) or requires further modification prior to their use in 3D cell cultures.^[^ ^222^ ^]^

To summarize, natural hydrogels currently serve as the primary matrices in the field of microphysiological engineering. However, their variability, combined with the constrained capacity for controlled modification (e.g., mechanical properties, binding motifs), restricts their suitability for advanced microphysiological models in their current state. Synthetic or engineered hydrogels, on the other hand, can be tailored to accommodate a range of biochemical and biomechanical properties, making them a promising alternative to naturally derived biomatrices. Among synthetic hydrogels, Poly(ethene glycol) (PEG) constitutes the most prominent matrix owing to its biocompatibility, inherent inertness as well as its modulus‐tunable backbone. With their high design flexibility, PEG hydrogels can be modified in numerous ways to accommodate various functional requirements, making them suitable for a broad spectrum of applications.^[^ ^215^ ^]^ *Papadimitriou* et al.,^[^ ^223^ ^]^ for example, developed a PEG‐heparin hydrogel to investigate the role of aβ_42_ on neural stem cell plasticity. *Brown* et al.,^[^ ^224^ ^]^ optimized the applicability of PEG hydrogels for engineering microvascular networks in vitro. Lowering the polymer and crosslinking density as well as reducing the hydrogel's swelling ratio, a limitation for microfluidic applications, resulted in the generation of homogenous vascular networks. The latter, however, remained non‐perfusable, necessitating further improvements prior to applications in neurovascular cultures.

Another notable approach among engineered hydrogels involves self‐assembling peptides or SAPs. SAPs describe short peptide sequences covalently modified to impart optimal hydrophilic–lipophilic balance for self‐assembly. Under physiological conditions, SAPs form fibrous nanostructures, that closely resemble the native ECM. By rational design, SAPs can be readily modified and decorated with functional and biological cues (e.g., ECM‐specific peptide sequences).^[^ ^225^ ^]^ RADA16 (PuraMatrix) the most prominent SAP, commercialized in 2002, has been shown to sustain 3D neuronal cultures for up to 4 weeks and demonstrated improved vascularization in vivo upon comparison with fibrin and collagen constructs.^[^ ^226^, ^227^ ^]^ Using PuraMatrix, *Zhang* et al.^[^ ^228^ ^]^ demonstrated the significance of 3D neuronal culture in recapitulating pathological phenotypes in vitro, as indicated by an aβ‐mediated redistribution of p21‐activated kinase in 3D compared to 2D. The application of SAPs in in vitro modeling, however, has remained limited.

Due to the lack of cell binding motifs in the native glycosaminoglycan, multiple strategies have focused on the development of engineered HA‐based hydrogels. Utilizing bioorthogonal copper‐free click chemistry, *Jury* et al.,^[^ ^229^ ^]^ for example, developed an HA‐laminin hydrogel, applicable to bioprinting. A recent study by *Isik* et al.^[^ ^230^ ^]^ reported a stiffness‐tunable (0.69 to 2.24 kPa) two‐component hydrogel combining HA and peptide amphiphiles applicable to cerebral organoid culture. The organoids embedded within the engineered matrix exhibited morphological and biomolecular signatures comparable to those embedded in Matrigel, highlighting the potential of engineered matrices as a viable alternative to the murine tumor‐derived ECM. The polysaccharide dextran, while distinct from HA, offers a biosimilar alternative with a bioorthogonal backbone applicable to mechanical tuning. *Stanton* et al.^[^ ^231^ ^]^ recently reported that the modification of dextran with cell‐degradable peptide sequences, RGD binding motifs, and versican facilitated the successful culture of a fully isogenic AD model comprising iPSC‐derived brain microvascular endothelial cells, pericytes, astrocytes, neurons, microglia, and oligodendrocytes. Neurovascular unit assembly was achieved at 90% to 100% ECM degradability, however, the engineered networks exhibited only limited perfusability.

Of note, one characteristic often overlooked in the engineering of hydrogels for modeling NDDs, is the matrices’ pore size. In vivo, aβ‐monomers (0.9 nm) and oligomers (1–20 nm) can easily traverse the neuropil.^[^ ^232^, ^233^ ^]^ However, native pore sizes have been estimated at 38–64 nm, which would locally restrict high molecular weight aβ‐oligomers, protofibrils, and fibrils (60–200 nm).^[^ ^234^, ^235^ ^]^ While the change in pore size in vitro upon cell culture remains unclear, most hydrogels typically exhibit significantly larger pore sizes ranging from 3–600 µm thus making them inadequate to replicate local protein aggregation upon perfusion.^[^ ^232^ ^]^

To conclude, existing hydrogel‐based matrices in and outside of MPS still fall short of replicating the intricate composition of the native ECM. Both natural and engineered hydrogels come with their own set of advantages and disadvantages, which must be carefully weighed based on the specific context of their application. Natural hydrogels, for example, exhibit favorable biodegradability, a multitude of cell‐interactive domains, and inherent stress‐relaxation behaviors (e.g., collagen, fibrin). While modifying the viscoelastic properties, an important parameter in the (patho)physiology of the native ECM, in natural hydrogels often accompanies changes in other key properties (e.g., stiffness), promising results have been achieved with alginate‐based matrices. *Charbonier* et al.^[^ ^236^ ^]^ recently published a methods paper summarizing three protocols that enable the viscoelastic tuning of alginate hydrogels. In contrast to natural hydrogels, which are limited by batch‐to‐batch variability and low customizability, synthetic or engineered hydrogels (e.g., PEG) stand out for their exceptional tunability and defined composition. In turn, synthetic materials lack tissue­specific structure and biochemical cues necessitating extensive modification. While the absence of modifications usually does not support essential cellular physiologies, precisely controlling this process favors their application for mechanistic studies. Furthermore, via rational design or the use of photoresponsive chemistries, engineered matrices can be tailored to induce spatiotemporal changes in vitro. Photopatterning has shown particular promise in the spatiotemporal modification of matrix stiffness and elasticity and has been employed, among others, to facilitate matrix modification at a single‐cell level, and to guide neural network growth in vitro.^[^ ^237^, ^238^ ^]^ For more information on modification strategies for spatiotemporal delivery in synthetic biomatrices please refer to the recent review by Blatchley and Anseth.^[^ ^239^ ^]^

However, the introduction of spatially and temporally confined dynamics is not limited to engineered or synthetic matrices. While spatially controlled deposition of native ECM constituents, for example, can be achieved through bioprinting – as demonstrated for decellularized ECM – spatiotemporal control in natural matrices can be obtained by interconnecting MPS to microfluidic gradient generators. For example, *Rifes* et al.^[^ ^240^ ^]^ modeled human neural tube formation in vitro by exposing embryonic stem cells to a WNT‐activating gradient, accomplished by a gradient generator situated upstream of the culture chamber.

In summary, adequately emulating the human brain in vitro necessitates a deeper understanding of the brain's intrinsic composition and its significance in (patho)physiological mechanisms. A variety of hydrogels have been developed so far, each presenting unique advantages and drawbacks. As such, upon advancing MPS of the human brain, the choice of biomaterial should be well thought out and tailored to the respective research question. This selection process should account for critical factors such as matrix composition, degradability, stiffness, elasticity, and pore size.

## 5 Local Microenvironments in NDDs

**Figure 6:**
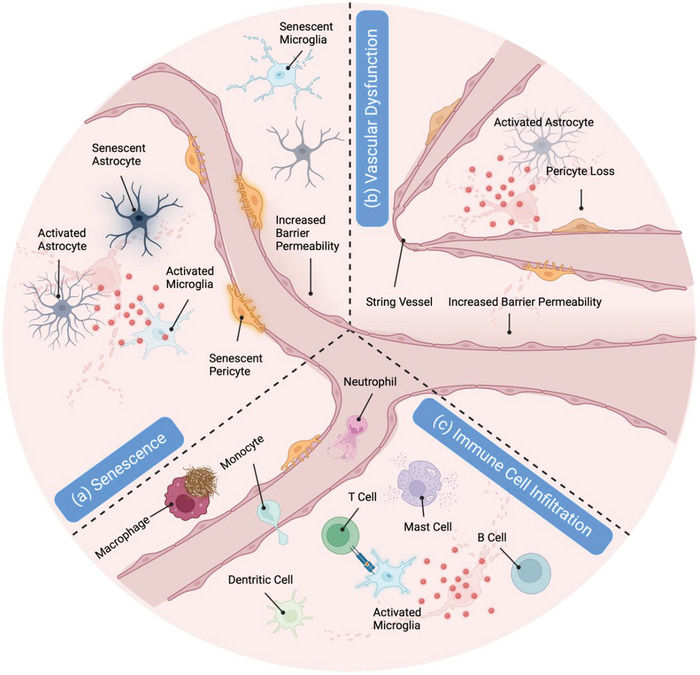
Graphical representation of three critical local microenvironments in NDDs: a) Graphical representation of local senescent microenvironments associated with NDDs highlighting the presence of senescent microglia, astrocytes, and pericytes. Alongside cellular senescence, local changes encompass astrocyte and microglial activation. b) Schematic depiction of local vascular dysfunction in NDDs characterized by the presence of impaired barrier integrity, string vessels, astrocyte activation, and pericyte loss. c) Schematic illustration of local immune cell infiltration reported in NDDs, encompassing *T*‐cells, mast cells, b cells, neutrophils, monocytes, and macrophages.

During NDD progression, the brain accumulates a set of conditions that increase cellular vulnerability such as aggregation of essential molecules, reduced energy metabolism and glutamate transport, as well as oxidative and mitochondrial damage. These conditions result in localized perturbations within the brain microenvironment, including alterations in biochemical and biophysical properties, and cellular functions.^[^ ^241^ ^]^ To illustrate, in AD, regions with aβ deposition exhibit shortened axon length and degeneration of neurites, resulting in a localized decline in synaptic connectivity.^[^ ^242^ ^]^ Moreover, studies have shown that localized changes in the immune microenvironment take place during late‐middle age (**Figure** **6**). While significant effort has been dedicated to investigating the impact of NDDs on the brain microenvironment, the role of these underlying changes in the onset and progression of the diseases still remains largely unknown.^[^ ^243^, ^244^, ^245^ ^]^ Studying the brain microenvironment during the onset and progression of brain disorders is key to identifying potential vulnerabilities and has implications for diagnosis and early intervention.^[^ ^246^ ^]^ In this section, we will describe local brain microenvironmental perturbations and discuss their role in NDDs. The ability of current MPS to replicate NDD‐associated localized microenvironmental changes will be discussed, alongside a critical evaluation of their inherent limitations.

### 5.1 Cellular Senescence in NDDs

Age is considered the primary risk factor for many NDDs including AD and PD, which manifest with greater prevalence in older individuals.^[^ ^247^ ^]^ Senescent cells, a common hallmark of aging, have been shown to accumulate over time at sites of NDD‐associated pathologies, actively promoting tissue degeneration (Figure 6a). This strongly suggests that senescent cells may promote cellular dysfunction and contribute to the development of disease pathology.^[^ ^248^, ^249^, ^250^, ^251^, ^252^ ^]^ Hallmarks of cellular senescence are sustained cell cycle arrest, telomeres attrition, increased DNA damage, increased protein oxidation, glycation, and misfolding, mitochondrial dysfunction, and expression of senescence‐associated secretory phenotypes, which consists of a variety of proteases, growth factors, and inflammatory cytokines.^[^ ^253^, ^254^, ^255^, ^256^ ^]^ The upregulation of these factors leads to low‐grade chronic inflammation termed inflammaging. In the brain, this state contributes to decreased synaptic density, resulting in a progression of cognitive impairment and memory loss in aged individuals.^[^ ^257^, ^258^, ^259^, ^260^ ^]^ Inflammation is a prevalent characteristic of NDDs, suggesting that aging and neurodegeneration share common proinflammatory pathways. It is important to note that senescence has been implicated in NDDs, independent of age.

A recent publication reported senescent pericytes and endothelial cells in close proximity to pre‐plaque sites in an AD mouse model. At these sites, BBB integrity was impaired, and endothelial cells showed a reduced expression of adherens junctions, implicating senescence in NDD‐associated vascular dysfunction.^[^ ^261^ ^]^ In addition, localized accumulation of senescent microglia has been observed in AD mice. This accumulation has been linked to the initiation of inflammation and neurotoxicity, contributing to the worsening and dissemination of the pathology.^[^ ^262^ ^]^ The presence of senescent microglia, characterized by telomere shortening, has also been confirmed in humans.^[^ ^263^, ^264^, ^265^, ^266^, ^267^, ^268^, ^269^, ^270^ ^]^ Astrocytes in AD‐affected patients have been shown to display increased DNA damage and overexpress inflammatory and senescent markers such as MMP1 and IL‐6.^[^ ^271^, ^272^, ^273^ ^]^ Senescent astrocytes have also been reported in other NDDs, such as PD and ALS, alongside the accumulation of senescent cells at sites of pathology.^[^ ^270^, ^274^ ^]^ For example, *Chinta* et al.,^[^ ^274^ ^]^ reported a significant increase in senescent markers in the substantia nigra pars compacta of PD patients compared to aged‐matched controls. Despite numerous studies, the intricate interplay between cellular senescence and the pathogenesis of NDDs remains inadequately understood. In recent years, there has been a growing interest in aging‐related research, particularly using models capable of replicating the intricate pathogenesis of age‐related diseases in vitro.^[^ ^275^, ^276^, ^277^ ^]^ For example, *Yamazaki* et al.^[^ ^278^ ^]^ employed a Transwell BBB model comprising endothelial cells and pericytes isolated from young and middle‐aged mice to study the effect of senescence on barrier integrity. Introducing senescent cells resulted in a significant increase in barrier permeability, confirming reported aging‐mediated alterations in BBB functionality.^[^ ^279^ ^]^

While animal cell sources are more accessible, key mediators of cellular senescence such as DNA damage and repair, telomere length, and telomerase activity, differ between humans and mice, underlining the importance of human‐based models in aging research.^[^ ^280^ ^]^

*Muwanigwa* et al.^[^ ^281^ ^]^ recently reported the presence of senescent astrocytes in human iPSC‐derived midbrain organoids carrying a triplication mutation of the a‐syn gene after 50 days of cultivation. Astrosenecence preceded dopaminergic neurodegeneration in vitro supporting the potential role of senescence in PD onset. *Barmpa* et al.,^[^ ^282^ ^]^ on the other hand, developed a human midbrain‐striatum assembloid model applicable to study PD‐associated phenotypes in the nigrostriatal pathway. Progerin‐overexpression within the model induced aging traits and accelerated the onset of PD phenotypes, underlining its potential for age‐related research. In OoC‐based models of the human brain, aging has been largely overlooked until now. Recently, however, *Ao* et al.^[^ ^283^ ^]^ reported a microfluidic model for the investigation of immune‐driven brain aging, a process by which immunosenescence drives system aging and the pathogenesis of age‐related diseases. The platform interfaces human brain organoids with flowing monocytes derived from young and aged donors. Aged monocytes displayed increased rates of infiltration and induced the expression of aging‐associated markers, suggesting a potential role in driving the aging process in the brain.

OoC technology and its ability to replicate complex microenvironments provide a powerful tool to emulate senescence‐associated perturbations in vitro. However, only a handful of studies have aimed at unraveling the relationship between aging and NDDs to date. One potential explanation is the lack of aged human cell sources. Studies that use established immortalized cell lines or primary cells derived from young donors are limited in replicating age‐related phenotypes. Therefore, it is crucial to utilize primary cells obtained from both young and elderly individuals. While this might be feasible, restricted availability and low yields render this approach unfavorable.^[^ ^284^ ^]^ To that end, future efforts should concentrate on employing techniques capable of reproducing age‐related characteristics in human cells including the introduction of established aging‐associated mechanisms, such as DNA damage and epigenetic alterations. Alternatively, inflammatory‐induced or replicative senescence protocols can be utilized.^[^ ^280^ ^]^ Moreover, transdifferentiation from peripheral blood mononuclear cells or fibroblasts has evolved into a powerful technique, allowing for the retention of aging features within differentiated neurons and glial cells, underscoring its significance as a valuable tool for developing in vitro models of aging.^[^ ^285^ ^]^ Next to investigating the role of senescent cells in NDDs, MPS can readily be employed to address the role of tissue–tissue interactions, e.g. the gut‐brain axis,^[^ ^286^ ^]^ in aging or age‐associated diseases, respectively. Furthermore, the ability to replicate complex interfaces in vitro will enable the investigation of senescent‐associated changes within the human neurovascular unit in NDDs.

To conclude, additional studies utilizing MPS are necessary to faithfully replicate aging‐associated microenvironments and to understand the implications of aging on human physiology and NDD pathology. The incorporation of senescent cells in MPS for drug screening could be pivotal for exploring the potential of senolytics in the treatment of NDDs and for recapitulating how age‐related cellular and physiological changes such as polypharmacy and multimorbidity affect the safety and efficacy of existing drugs.^[^ ^287^, ^288^ ^]^

### 5.2 Vascular Dysfunction in NDDs

Vascular pathology in the brain is often concurrent with NDDs. BBB dysfunction has been established through neuroimaging, postmortem tissue analysis, and examination of cerebrospinal fluid samples as an early (patho)physiological event in the onset of NDDs. Consequently, BBB dysfunction has been recognized as an early biomarker in NDDs.^[^ ^289^, ^290^ ^]^ The function of the BBB is regulated by various cell types, such as astrocytes, pericytes, neurons, and microglia, overall composing the neurovascular unit.^[^ ^291^, ^292^ ^]^ This specific microenvironment maintains the integrity of the BBB and regulates the supply of cerebral blood flow (CBF) in the capillaries, both key to maintaining normal brain function.^[^ ^293^ ^]^ In the neurovascular unit, the BBB acts as a functional selective barrier, protecting the brain from pathogenic infections and harmful substances entering from the bloodstream. Numerous studies have shown the implication of the BBB in the pathogenesis of NDDs such as AD, PD, HD, and ALS.^[^ ^294^, ^295^, ^296^, ^297^ ^]^ Evidence highlights that brain vascular dysfunction appears before the symptomatic onset of NDDs. As NDDs progress, changes in the vascular morphology and the cells of the neurovascular unit emerge, altering CBF and vascular integrity, thus accelerating the severity of the disease.^[^ ^298^, ^299^, ^300^, ^301^, ^302^, ^303^ ^]^ Dysfunction of the BBB includes changes in permeability, morphology, angiogenesis, vascular regression, and dysregulation of endothelial cell transporter proteins and receptors (Figure 6b).

In AD, BBB disruption begins with changes in permeability, associated with a reduction of pericyte density, and a decrease in tight junction proteins in endothelial cells, such as claudins, occludins, and ZO‐1.^[^ ^290^, ^304^, ^305^ ^]^ Pericytes cover the abluminal surface of the blood vessels and control and maintain BBB function and diameter.^[^ ^306^, ^307^ ^]^ A reduction in pericyte number and density was shown to increase expression levels of aβ and p‐tau, along with compromised BBB integrity and increased permeability, thus aggravating AD progression.^[^ ^303^, ^308^, ^309^, ^310^ ^]^ BBB damage is associated with aβ deposition in the vascular bed, which induces proinflammatory cytokine release from microglia resulting in neurotoxicity.^[^ ^303^, ^311^ ^]^ Morphological changes in astrocytes were observed in the vicinity of aβ deposition.^[^ ^312^ ^]^ Astrocytes are a key component of the BBB and are crucial for its integrity since they facilitate the formation of endothelial tight junctions. Astrocytes in AD patients were morphologically different from those of the healthy control group, and this difference was correlated with an accelerated BBB breakdown.^[^ ^313^, ^314^ ^]^ A primary function of the BBB is to facilitate the removal of toxins and waste from the brain parenchyma through transporter proteins and receptors. These are also crucial to aβ transport across the BBB and are altered in AD, leading to further aβ accumulation, BBB dysregulation, and loss of homeostasis. Endothelial cells of AD patients display decreased expression of P‐glycoprotein (P‐gP) and low‐density lipoprotein receptor‐related protein (LRP1), which hinder aβ clearance from the brain. This process is aggravated by an increase in the expression of the receptor for advanced glycation end products (RAGE), transporting aβ from the circulation to the brain.^[^ ^292^, ^315^, ^316^, ^317^, ^318^, ^319^, ^320^ ^]^ In addition, lower levels of the transporter protein GLUT1 are found in AD patients, which are associated with vascular degeneration and reduction in glucose levels.^[^ ^321^ ^]^ More details on physiological transporter mechanisms can be found in Section 5.4. Histopathological examinations have shown changes and remodeling of the brain microvasculature of AD patients. Common morphological changes include the presence of fragmented vessels, increased thinning of microvessels or so‐called string vessels, glomerular loop formation, increased vessel tortuosity, and angiogenesis, significantly affecting brain homeostasis.^[^ ^322^, ^323^, ^324^, ^325^, ^326^, ^327^ ^]^ A higher number of new and fenestrated blood vessels have been observed in the postmortem brain of patients.^[^ ^303^ ^]^ Impaired BBB function is also pivotal in the pathological mechanisms of PD, where α‐syn deposition is associated with increased BBB permeability.^[^ ^328^ ^]^ BBB disruption is promoted by pericyte activation and proinflammatory cytokine production from astrocytes and microglia.^[^ ^329^ ^]^ This induces a lower expression of tight junction proteins as well as deregulation of transporter proteins and receptors.^[^ ^328^ ^]^ Similarly to AD, PD patients have more string vessels and less P‐gP, causing a decreased efflux membrane transport and accumulation of α‐syn.^[^ ^330^, ^331^, ^332^ ^]^ PD patients show microvasculature remodeling including angiogenesis.^[^ ^333^ ^]^ Although fewer studies have been performed on ALS and HD, BBB dysfunction was reported in patients. Pathological changes include a reduced expression of tight junction proteins, reduction in astrocyte numbers, capillary structural impairment, increased blood vessel density, decreased expression of GLUT‐alpha, as well as overexpression of P‐gP.^[^ ^289^, ^290^, ^334^, ^335^, ^336^ ^]^

During the last 10 years, significant progress has been made in emulating the human BBB in vitro *(also see Introduction)*. However, only recently, BBB models were expanded by neurons and microglia, thus representing well‐defined features of the brain, such as the neurovascular unit. *Shin* et al.^[^ ^19^ ^]^ developed a microfluidic chip to model the neurovascular unit and investigate BBB dysfunction in AD. Accumulation of aβ and p‐tau was observed in the brain channel of the platform, while BBB disruption and decreased expression of tight junctions were reported in the vascular compartment of the device. Inhibiting aβ secretion using a β‐secretase inhibitor significantly reduced barrier permeability and increased claudin‐5 expression, implicating aβ in the observed vascular dysfunction. *Jang* et al.^[^ ^337^ ^]^ employed a coculture model of a singular lumen of primary human microvascular endothelial cells and neural progenitor cells to investigate the effect of hyperglycemia on the development of AD‐associated phenotypes. Hyperglycemia, which was induced by 25 mM D‐glucose, resulted in brain vascular dysfunction, including downregulation of ZO‐1 and VE‐cadherin, aβ and p‐tau accumulation, and a 30% decrease in neuronal viability upon the addition of exogenous aβ. An alternative approach to investigating vascular dysfunction in AD was reported by *Ko* et al.^[^ ^25^ ^]^ Coculturing vascular networks of the human BBB in close proximity to aβ overexpressing neuronal tissues elicited significant morphological changes encompassing reduced vessel length, number of branch points, as well as vessel diameters. Furthermore, next to vascular aβ deposition, a significant increase in barrier permeability was observed after seven days of microfluidic co‐culture. *Pediaditakis* et al.^[^ ^338^ ^]^ employed a neurovascular unit chip incorporating brain endothelial cells, pericytes, astrocytes, microglia, and cortical neurons to investigate BBB dysfunction in the context of neuroinflammation. Inflammation was induced by the addition of TNF‐ α within both luminal and abluminal compartments of the microfluidic platform. TNF‐ α treatment resulted in BBB disruption, a substantial decrease of GLUT‐1, astrocyte, and microglial activation, as well as increased cytokine release. The same platform was employed to investigate neurovascular dysfunction in the context of PD. Exogenous addition of α‐syn fibrils for a period of five days significantly increased barrier permeability and resulted in LRP1 and ABCB1 upregulation.^[^ ^18^ ^]^

While significant progress has been made in developing OoC platforms for studying NDD‐associated vascular dysfunction, their applicability currently remains restricted to AD and PD. Replacing current cell sources with iPSC‐derived cells will broaden their applicability to other NDDs and enable the development of patient‐specific models. Moreover, leveraging patient‐specific cells will aid in elucidating the underlying mechanisms of both familial and idiopathic NDDs within the context of vascular dysfunction. To investigate pathological changes including vascular remodeling and regression, self‐assembled networks will become indispensable. Extended culture periods, tailored to the timeframes required for vascular remodeling and progressive degeneration, however, will require continuous perfusion, necessitating the use of fluid circulation setups. Given the importance of neurovascular unit models in drug screening, establishing standardized and effective validation strategies for in vitro models will become paramount for a successful clinical translation. Herein, the identification of therapeutic and diagnostic NDD markers will be essential. Lastly, a thorough characterization of endothelial cell transporters will be required to ensure physiological protein expression levels in drug screening studies.

#### 5.2.1 Transporters of the BBB


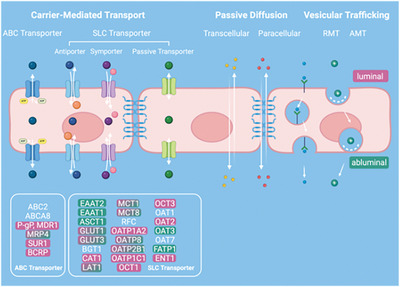
The BBB is recognized as a dynamic interface with specific physiological roles, facilitating selective transport of substances from the blood into the brain and actively removing compounds from the brain back to the luminal side.^[^ ^339^ ^]^ The different transporter mechanisms of the BBB can be classified as carrier‐mediated transport (CMT), passive diffusion, and vesicular transport.^[^ ^340^ ^]^ Within the CMT, ABC transporters are responsible for regulating the active efflux of potentially harmful substances employing ATP hydrolysis. Solute carrier transporters (SLC) mediate the uptake and efflux of brain nutrients.^[^ ^341^ ^]^ Passive diffusion through the BBB occurs via two pathways: transcellular diffusion (through the cell), in which small lipophilic molecules move through the luminal membrane, cytosol, nucleus, and abluminal membrane reaching the CNS, and paracellular (between adjacent cells), in which ions and solutes travel using concentration gradients.^[^ ^342^ ^]^ Vesicular trafficking is predominantly a transcellular mechanism. Large molecules are actively transported from the luminal toward the abluminal side through receptor‐mediated transcytosis (RMT) or adsorptive‐mediated transcytosis (AMT). In RMT, ligands bind to specific receptors situated on the luminal surface of the endothelial cells. Upon binding, the ligands are internalized and then transported into the brain. In AMT, electrostatically charged molecules bind to anionic molecules of the luminal surface of the endothelial cells, they are internalized in vesicles and transported across the abluminal membrane.^[^ ^341^, ^343^ ^]^

### 5.3 Immune Cell Infiltration in NDDs

For decades, the prevailing notion portrayed the brain as self‐sufficient and isolated from peripheral immune activity, based on the assumption that resident immune cells, such as microglia, and the presence of barrier systems within the brain sufficed for neuroprotection, maintenance, and repair.^[^ ^41^ ^]^ However, increasing evidence in recent years has implicated infiltrating non‐resident immune cells in the pathogenesis of NDDs.^[^ ^41^, ^344^, ^345^, ^346^ ^]^ It is now known that changes in the brain immune microenvironment, such as variation in the density and function of immune cells and alterations in immunological homeostasis occur even before the onset of NDD symptoms.^[^ ^346^, ^347^ ^]^ Infiltrating immune cells comprising antigen‐specific cells such as *T*‐cells, *B*‐cells, dendritic cells, monocytes, and non‐specific granulocytes such as mast cells, penetrate the CNS through different barriers (Figure 6c). Next to the NDD‐associated breakdown of the BBB, which constitutes the most common infiltration route, immune cells have been shown to transmigrate through the blood‐leptomeningeal barrier and the blood‐CSF barrier.^[^ ^348^, ^349^, ^350^, ^351^ ^]^ The recruitment of infiltrating cells initiates with the activation of microglia and astrocytes, which release chemokines and cytokines into the bloodstream and upregulate the expression of cellular adhesion molecules, thereby inducing leukocyte transmigration into the parenchyma.^[^ ^351^, ^352^ ^]^ While the activation of the brain's intrinsic immune system and the subsequent infiltration of peripheral immune cells are commonly thought to maintain tissue homeostasis in vivo, the presence of peripheral immune cells in the CNS is believed to be linked to NDD onset.^[^ ^263^ ^]^ *T*‐cells, specifically CD4+ helper T and CD8+ cytotoxic *T*‐cells were found in the parenchyma of AD and PD patients.^[^ ^353^, ^354^ ^]^ The number of CD4+ T cells in the CSF of mice and patients with inflammatory diseases increases significantly, promoting the infiltration of monocytes and, thus, neurotoxicity.^[^ ^346^, ^355^, ^356^ ^]^ CD8+ *T*‐cells, too, have also been shown to induce neurodegeneration in the neuropil through MHC‐I‐mediated activation.^[^ ^355^ ^]^ Various B cell clones were found in different brain compartments, confirming their ability to cross CNS barriers. To illustrate, elevated B cell numbers have been reported in the parenchyma of AD patients, where they were linked to local aβ plaque deposition. However, our understanding of the mechanisms by which B cells can traverse CNS barriers remains limited.^[^ ^357^, ^358^ ^]^ In the brains of healthy patients, mast cells are localized in the meningeal layers and the abluminal perivascular areas of the brain parenchyma. In AD, an increase in the number of mast cells is found in proximity to aβ plaques, while in PD patients, mast cells induce microglia activation and release proinflammatory molecules that promote inflammation and neurodegeneration.^[^ ^345^, ^346^, ^359^, ^360^, ^361^, ^362^ ^]^ Dendritic cells are recruited into the CNS by a variety of chemokines and receptors, and their numbers have been shown to increase alongside brain inflammation in the case of both PD and AD, suggesting a potential role in the pathogenesis of NDDs.^[^ ^346^, ^363^, ^364^, ^365^, ^366^ ^]^

Changes in the quantity and functionality of immune cells within the CSF can be leveraged as an early non‐invasive diagnosis tool for NDDs as well as a potential therapeutic target.^[^ ^346^ ^]^ In animal models, therapies directed toward *T*‐cells, B cells, and dendritic cells have shown favorable results. Since *B*‐cells are responsible for the production of disease‐specific antibody titers, emerging immunotherapies are aimed at depleting *B*‐cells in humans.^[^ ^367^ ^]^ Overall, these results underline the necessity of investigating the complex relationship between the immune microenvironment and the onset of NDDs.^[^ ^264^, ^346^, ^368^, ^369^ ^]^ In vitro models able to replicate the functionality of the human brain immune system are essential for addressing these knowledge gaps and facilitating clinical translation.^[^ ^370^ ^]^

Several MPS models have been developed to investigate immune cell penetration through the BBB in response to inflammation. The majority of these platforms utilize a membrane‐based setup to monitor immune cell transmigration across the BBB.^[^ ^220^, ^370^, ^371^, ^372^, ^373^, ^374^, ^375^, ^376^ ^]^ Alternatively, a more physiologically relevant approach for studying immune cell infiltration in vitro involves employing self‐assembled networks of the BBB, which have already shown success in studying cancer cell extravasation.^[^ ^377^ ^]^

As of now, immune cell studies have been limited to the BBB, neglecting other existing barriers through which immune cells infiltrate. A potential setup that could be applicable to study immune cell infiltration through the blood‐CSF barrier was developed by *Lim* et al.^[^ ^80^ ^]^ In their study, the authors perfused macrophages and tumor cells through a vascular network cultured adjacent to an engineered choroidal tissue. Dynamic culture improved tissue mimicry, increased macrophage motility, and demonstrated enhanced anti‐cancer efficacy through synergetic effects between immune cells and administered Trastuzumab. *T* and *B*‐cells have also been located in lymphatic vessels, suggesting a role of meningeal lymphatics in the trafficking of immune cells.^[^ ^32^, ^156^ ^]^ A promising platform to investigate the underlying mechanisms of immune cell trafficking was designed by *Serrano* et al.^[^ ^82^ ^]^ In their work, the authors developed an in vitro lymphatic system recapitulating in vivo‐like functionality and morphology. Using this setup, *Serrano* et al. investigated the immune response of the model by introducing human peripheral blood mononuclear cells comprising a broad population of immune cells, such as *T*‐cells, dendritic cells, macrophages, and natural killer cells. The authors were able to monitor the infiltration of the immune cells into the lymphatic gel region following a gradient of TNF‐α.

However, while MPS hold great potential in investigating immune cell infiltration, very little has been done in the context of NDDs. Recently, *Jorfi* et al.^[^ ^139^ ^]^ reported a 3D human neuroimmune axis model of AD comprising stem cell‐derived neurons, astrocytes, microglia, and human peripheral immune cells. Similar to the model by *Park* et al.,^[^ ^26^ ^]^ the device employs radially arranged microchannels to generate gradients of CNS‐secreted soluble factors and to spatially separate astrocytes, neurons, and microglia from peripheral immune cells. AD co‐cultures showed an increase in CD8+ *T*‐cell infiltration that led to microglial activation and exacerbation of AD neurodegeneration. Using this model, the authors demonstrated the key role of the CXCL10‐CXCR3 in regulating *T*‐cell infiltration, identifying the chemokine as a potential new therapeutic target.

OoC platforms provide a powerful tool to investigate the crosstalk between immune cells and cells of the local brain microenvironment, essential to uncovering disease mechanisms and potential therapeutic targets. While MPS have been employed to investigate immune cell infiltration in vitro, limited progress has been made in the context of NDDs. The lack of microphysiological immune models might be associated with issues in distinguishing self versus non‐self immunity.^[^ ^370^ ^]^ Thus, by co‐culturing cells of different donors, immune cells can be activated by HLA heterogeneity. Avoiding immune cell activation in MPS consequently necessitates the use of isogenic models. In the last few years, an increasing number of immune cell types, including macrophages, dendritic cells, *T*‐cells, mast cells, B cells, microglia, and neutrophils have been successfully derived from iPSCs, making fully isogenic models a concrete possibility.^[^ ^370^, ^378^, ^379^, ^380^, ^381^, ^382^ ^]^ Furthermore, this approach would allow for the development of patient‐specific models, enabling the investigation of idiopathic NDDs and paving the way for personalized medicine. Prior to their use, however, it will be imperative to evaluate the reprogrammed cells’ capability to recapitulate physiological functions and phenotypes.

## 6 Balancing Complexity and Robustness in MPS

In vitro models of the human brain have evolved enormously over the last decade, enabling the recapitulation of complex aspects of the native microenvironment. OoC platforms, specifically, have been able to mimic (patho)physiological tissue niches with unprecedented fidelity. The combination of elaborate platform designs with the induction of biophysical cues and the spatially controlled arrangement of various cell types and matrices favors the engineering of highly complex tissue mimics in vitro. While increasing model complexity will help in faithfully replicating (patho)physiological microenvironments in vitro, it risks potential compromises in model control and reproducibility.^[^ ^383^ ^]^ Thus, finding a fine balance between MPS complexity and robustness will become essential to ensure translational value in future OoC models.

A primary factor contributing to the challenge of reproducibility in complex MPS originates from the choice of cell source, underlining the importance of standardization in cell culture. While primary cells most closely represent the human tissue in vivo, aside from finite lifespans, comparability among primary cells is limited by donor and subculture variability. Cell lines, on the other hand, characterized by their unlimited lifespan and low costs, suffer from low biological relevance and genetic instability, rendering them unsuitable for potential drug studies.^[^ ^384^, ^385^ ^]^ Over the last decade, iPSCs have demonstrated tremendous potential due to their ethical and unlimited sourcing, their ability to replicate pathological phenotypes on a patient‐specific basis, as well as their ability to adopt virtually any cellular fate.^[^ ^386^ ^]^ However, iPSCs, too, display donor, inter‐clonal, and intra‐clonal variability, hampering comparability.^[^ ^387^ ^]^ To illustrate, a study by *Volpato* et al.^[^ ^388^ ^]^ assessing inter‐laboratory variation reported a discrepancy of 7457 differentially expressed genes when comparing the transcriptomic data from cortical neurons derived from two iPSC lines differentiated at two different sites employing the same protocol. Furthermore, erased aging signatures, an intrinsic feature of iPSCs, must be considered when modeling age‐associated pathologies such as NDDs in vitro, where iPSCs may predominantly reflect the early stages of the disease.^[^ ^389^ ^]^ Recognizing the inherent variability in iPSCs, however, numerous guidelines have been published and made publicly available in recent years to ensure optimal reproducibility.^[^ ^390^, ^391^, ^392^ ^]^ This is of particular importance, considering the vast potential of iPSCs for advancing current MPS.

The ability to generate autologous models using iPSCs is particularly important in the context of immune‐competent models of the brain, given the poor predictive validity reported for animal models in immunology.^[^ ^393^ ^]^ While autologous MPS models, excluding organoids, are still limited in number, *Stanton* et al.^[^ ^231^ ^]^ recently reported the first AD‐patient‐specific in vitro model of the neurovascular unit incorporating brain microvascular endothelial cells, pericytes, astrocytes, neurons, oligodendrocytes, as well as microglia by employing a dextran‐based hydrogel. Furthermore, the superior tissue mimicry of iPSC‐derived organoids renders them a powerful tool to increase tissue complexity within OoC systems.^[^ ^394^ ^]^ For example, patient‐specific organoids can be integrated into existing platforms to study the complex cellular interplay in vascular dysfunction. Here, platforms such as those reported by *Ko* et al.^[^ ^25^ ^]^ and *Nashimoto* et al.^[^ ^395^, ^396^ ^]^ featuring the co‐culture of self‐assembled vascular networks with tumor and neuronal spheroids, respectively, hold great promise. To investigate the complex brain–immune system interactions, brain organoids incorporating microglia could be introduced in platforms with circulating immune cells.^[^ ^283^, ^397^ ^]^ While the intrinsic variance in iPSC‐derived organoids may contribute to MPS variability, significant advances have been made in the development of robust organoid production pipelines.^[^ ^398^ ^]^ These advances should be taken into consideration in future models. An alternative approach to increasing complexity in MPS is the use of interconnected platforms. While this might be helpful in breaking down the complex structure of the human brain itself, it is of specific importance in the context of studying systemic effects (e.g., gut‐brain axis^[^ ^399^ ^]^), which is still largely overlooked in current models of NDDs.

To ensure that variability in MPS of increasing complexity solely originates from biological variation, it is imperative to establish clear standards. To address the unmet need for standardization in OoC technology, currently hindering the successful adoption and integration of MPS in a clinical setting, several initiatives have been implemented over the last few years. Next to cell source, standardization needs to be addressed on multiple fronts, including, among others, the selection of materials, assays, and biomarkers, interoperability, and control systems, as well as data management.^[^ ^400^, ^401^, ^402^, ^403^ ^]^ Furthermore, as MPS models progress in complexity, there is an increasing demand to integrate metrology into the system at both device and system levels.

Multiparametric sensing strategies, combined with MPS, are increasingly important for real‐time monitoring of tissue functionality, microenvironmental parameters, and the progression of pathological phenotypes. These strategies will help investigate and unravel mechanisms that may otherwise be missed using endpoint analysis alone. Despite the availability of numerous non‐invasive sensing strategies, such as electrical, electrochemical, and optical sensors, which can be integrated on‐ and off‐chip, the majority of in vitro NDD models still lack essential noninvasive sensing strategies to capture dynamic pathological changes.^[^ ^404^ ^]^

It is important to note, that while increasing complexity will help in the recapitulation of intricate microenvironments, MPS still represent an approximation of human physiology. Thus, drawing conclusions on the in vivo situation and overcoming experimental limitations associated with MPS will require the assistance of alternative approaches. Computational models have already been successfully employed to model and predict pathological changes in NDDs, making them a promising complementary strategy to OoC technology. Numerous computational models have been built to investigate different aspects of AD,^[^ ^405^, ^406^, ^407^ ^]^ PD,^[^ ^408^, ^409^ ^]^ HD,^[^ ^410^ ^]^ and ALS.^[^ ^411^, ^412^ ^]^ *Chamberland* et al.,^[^ ^405^ ^]^ for example, developed a multiscale mathematical model comprising a system of ordinary differential equations that utilizes various inputs from patients of different ages affected by AD. Their model effectively described AD progression in relation to brain aging, and explored the influence of type 2 diabetes on AD. Moreover, existing computational models of the lymphatic system could be combined with the abovementioned NDD model to investigate changes in the clearance of toxic molecules during disease progression.^[^ ^413^ ^]^ Among computational models, digital twins have emerged as a promising approach. The underlying concept involves connecting a physical model to its identical virtual counterpart using extensive patient data acquired from medical examinations and analyses.^[^ ^414^ ^]^ The digital model can integrate all types of variables relevant to pathogenesis and obtain virtual replicas of tissues, organs, or even entire organisms. This approach holds the potential for unraveling complex (patho)physiological processes and revolutionizing precision medicine by simulating therapies and prognosis of disease progression.^[^ ^414^ ^]^ *Cen* et al.^[^ ^415^ ^]^ built a digital twin of multiple sclerosis that is able to estimate the age of brain atrophy onset by using brain MRI scans as input. However, the use of digital twins in healthcare is still in its infancy, and challenges related to data quality and integration, computational power demand, and patient privacy and security still need to be addressed.^[^ ^414^ ^]^ To date, applications of digital twins in disease modeling have been largely limited to computational models that mimic aspects of human pathology, often in the context of patient‐specific models. But given the potential for increased complexity in MPS, there is also value in creating a digital twin of an MPS wherein the parameters of the digital twin are obtained from simpler, more reproducible, and easily controlled systems and then used as a basis for creating a more complex system, such as a multi‐organ MPS. The advantages lie in the ability to perform systematic parameter sensitivity analysis and test hypotheses that can subsequently be validated in either MPS or clinical studies.

OoC technology has advanced significantly in recent years, achieving remarkable levels of biomimicry. However, these systems must also be robust, with established standardization protocols to guarantee reproducibility. In this context, sensing strategies will be crucial, as non‐invasive monitoring can generate valuable datasets and maintain proper system functionality. Moreover, to close the gap between MPS and in vivo microenvironments, interdisciplinary approaches such as in silico models offer promising solutions.

## 7 Conclusion

To conclude, significant progress has been made in replicating complex physiological tissue niches of the human brain employing OoC platforms. This progress includes the replication of key NDD‐associated phenotypes encompassing, among others, abluminal aβ deposition, parenchymal aβ and p‐tau accumulation, vascular dysfunction, mitochondrial impairment, neurodegeneration, neuroinflammation, and microglial recruitment. While current models are predominantly restricted to the study of AD and PD, many platforms can readily be adapted to investigate other NDDs including ALS and HD.

Existing models, however, still fall short of addressing critical aspects that play a key role in NDD onset and progression. The distinct architecture and organization of the brain's clearance system, along with its emerging role in NDDs, underscores the importance of its inclusion in future MPS. Careful consideration of suitable biomaterials is essential, and this assessment should be contextual, enabling a balance between achieving optimal biomimicry and minimizing variability. In light of mounting evidence implicating the immune system in NDD pathology, future MPS should incorporate both innate and non‐resident immune cells to accurately model the intricate mechanisms underlying neurodegeneration. Furthermore, upcoming studies should leverage the ability of OoC technology to replicate complex dynamic changes observed in the brain microenvironment including local accumulation of senescent cells, infiltrating immune cells, and vascular dysfunction. Alternative strategies, such as in silico models and digital twin technology, will help in the effort to augment model complexity by overcoming experimental limitations. Alongside an increase in model complexity, ascertaining reproducibility will become especially important. Employing non‐invasive sensing strategies, coupled with the use of robust cell sources and protocols will help in this endeavor. Taking these factors into account will advance the in vitro modeling of NDDs in future MPS and enhance their translational relevance in clinical applications.

## Conflict of Interest

R.D.K. is the co‐founder of and holds a significant financial interest in AIM Biotech, a company that produces microfluidic devices. He also receives research support from Amgen, Daiichi‐Sankyo, Novartis, Boehringer Ingelheim, AbbVie, Takeda, Eisai, Visterra, EMD Serono and Roche.

## Author Contributions

F.M.P. and S.S. contributed equally to this work. FMP performed conceptualization and wrote the final manuscript. SS performed conceptualization and graphical illustration, and wrote the final manuscript. RDK performed conceptualization and wrote the final manuscript. All authors reviewed and approved the final manuscript.
